# Synthetic α‐Helical Peptides as Potential Inhibitors of the ACE2 SARS‐CoV‐2 Interaction

**DOI:** 10.1002/cbic.202200372

**Published:** 2022-07-14

**Authors:** Pascal M. Engelhardt, Sebastián Florez‐Rueda, Marco Drexelius, Jörg‐Martin Neudörfl, Daniel Lauster, Christian P. R. Hackenberger, Ronald Kühne, Ines Neundorf, Hans‐Günther Schmalz

**Affiliations:** ^1^ Department of Chemistry University of Cologne Greinstrasse 4 50939 Cologne Germany; ^2^ Department of Chemistry University of Cologne Zülpicher Straße 47a 50674 Cologne Germany; ^3^ Leibniz-Forschungsinstitut für Molekulare Pharmakologie (FMP) Robert-Rössle-Strasse 10 13125 Berlin Germany; ^4^ Freie Universität Berlin Institut für Biochemie und Chemie Arnimallee 22 14195 Berlin Germany

**Keywords:** CD spectroscopy, peptides, protein-protein interactions, SARS-CoV-2, secondary structures

## Abstract

During viral cell entry, the spike protein of SARS‐CoV‐2 binds to the α1‐helix motif of human angiotensin‐converting enzyme 2 (ACE2). Thus, alpha‐helical peptides mimicking this motif may serve as inhibitors of viral cell entry. For this purpose, we employed the rigidified diproline‐derived module **ProM‐5** to induce α‐helicity in short peptide sequences inspired by the ACE2 α1‐helix. Starting with Ac‐QAKTFLDKFNHEAEDLFYQ‐NH_2_ as a relevant section of α1, a series of peptides, *N‐*capped with either Ac‐βHAsp‐[**ProM‐5**] or Ac‐βHAsp‐PP, were prepared and their α‐helicities were investigated. While **ProM‐5** clearly showed a pronounced effect, an even increased degree of helicity (up to 63 %) was observed in sequences in which non‐binding amino acids were replaced by alanine. The binding affinities of the peptides towards the spike protein, as determined by means of microscale thermophoresis (MST), revealed only a subtle influence of the α‐helical content and, noteworthy, led to the identification of an Ac‐βHAsp‐PP‐capped peptide displaying a very strong binding affinity (K_D_=62 nM).

## Graphical abstract

**The** ***N*** **‐cap makes the difference**: Aiming at the development of potential COVID‐19 drugs distorting the interaction between the SARS‐CoV‐2 spike protein and human ACE2, a series of *N*‐capped peptides derived from the α1‐helix of ACE2 were prepared to increase the α‐helical content. This led to the discovery of a comparably short peptide binding to the spike protein with high affinity (K_D_=62 nM).

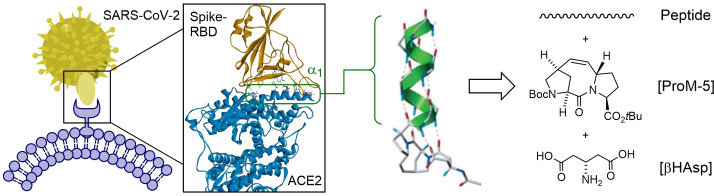

## Introduction

**Figure 1:**
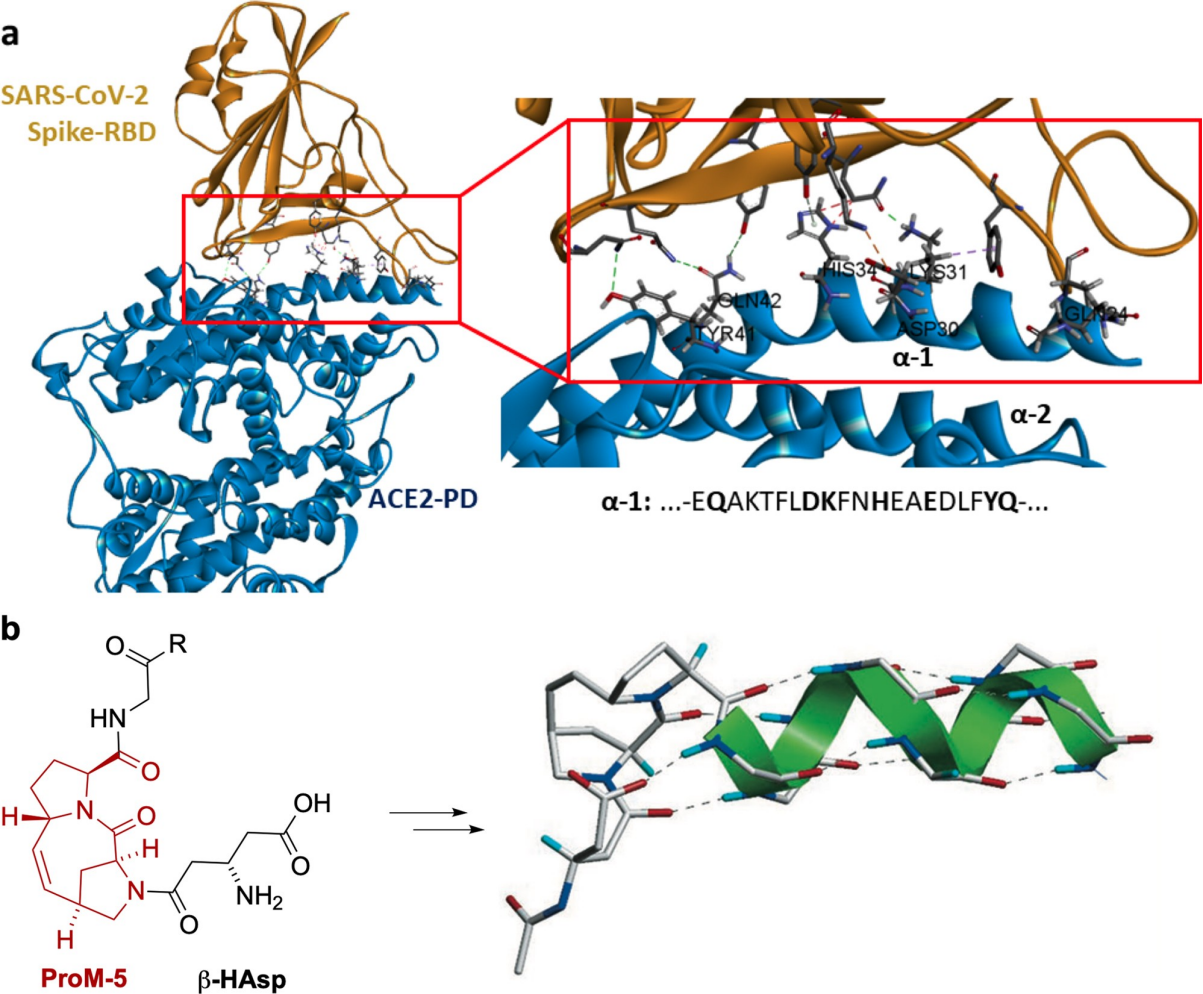
a: Structure of spike‐RBD co‐crystallized with ACE2‐PD and a magnification of the binding region (adapted from Yan *et al*.^[5]^). The sequence of the main binding motif of α1 is given with binding‐relevant amino acids highlighted in bold. b: The use of Ac[**βHAsp**]‐[**ProM‐5**] as an *N*‐cap induces α‐helix nucleation in short peptides (adapted from Hack *et al*.^[6a]^).

Two years after the discovery of the novel SARS CoV‐2 coronavirus, the pandemic resulting from the associated coronavirus disease (COVID‐19) is still a major concern for the global community, with over 400 million confirmed cases and nearly 6 million deaths by early 2022.^[1]^ Although the development of vaccines has brought some relief, the virus continues to spread.^[2]^ Therefore, repurposing known drugs and developing new therapeutic agents against the disease is an important and rapidly advancing area of research.^[3]^ In early 2020, it was discovered that cell entry of SARS‐CoV‐2 proceeds via primary binding of the viral spike protein to human angiotensin‐converting enzyme 2 (ACE2).^[4]^ In addition, crystal structures provided more detailed insights into the specific interaction of the peptidase domain (PD) of ACE2 with the receptor binding domain (RBD) of the spike protein.^[5]^ In this context, the major amino acids involved in the binding process appear to be located at an alpha helix motif called α1 (Figure 1a). Peptides mimicking the α1‐helix could therefore serve as synthetic small molecule inhibitors for the spike protein. However, one problem with such approaches is that for smaller peptides, the secondary structure stabilized in the protein by additional interactions is often lost.

In 2013, we presented a method for increasing the helix content of short peptide sequences by using the proline‐derived module **ProM‐5** as an *N*‐cap.^[6]^ This molecule, which represents a pair of two proline units covalently linked by a vinylidene bridge, is conformationally locked and able to nucleate the formation of an α‐helix secondary structure when combined with β‐homoaspartic acid (**βHAsp**) (Figure 1b).^[6a]^ While the success of this approach was demonstrated by CD spectroscopy using model peptides in the past, the question remained whether peptide sequences derived from natural α‐helical protein substructures could be also be improved this way with respect to their desired biological (inhibitory) effects.

Thus, as a relevant challenge, we decided to apply our concept to the development of potential inhibitors of the SARS‐CoV‐2 ACE2 interaction by employing peptides derived from the α1‐helix. It should be noted that other groups have also recently reported the development of α1‐derived peptides as potential spike‐binding molecules,[^7^, ^8^] also stressing the importance of the α‐helical preorganization.^[8]^ However, the use of helicity‐inducing *N*‐caps, which are in the focus of the present study, has so far never been investigated in this context.

## Results and Discussion

**Scheme 1:**
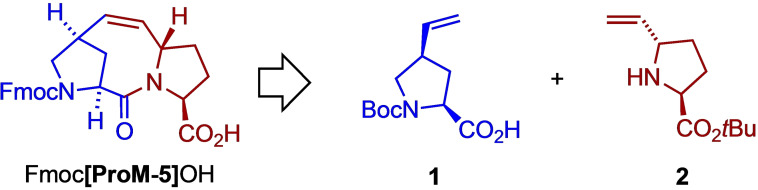
Retrosynthetic analysis of Fmoc[**ProM‐5**]OH leading to the vinylproline building blocks **1** and **2**.

We started our investigation with the synthesis of **ProM‐5** following our established strategy (Scheme 1) which is based on the coupling of the vinyl‐proline derivatives **1** and **2** and subsequent ring closing metathesis.

**Scheme 2:**
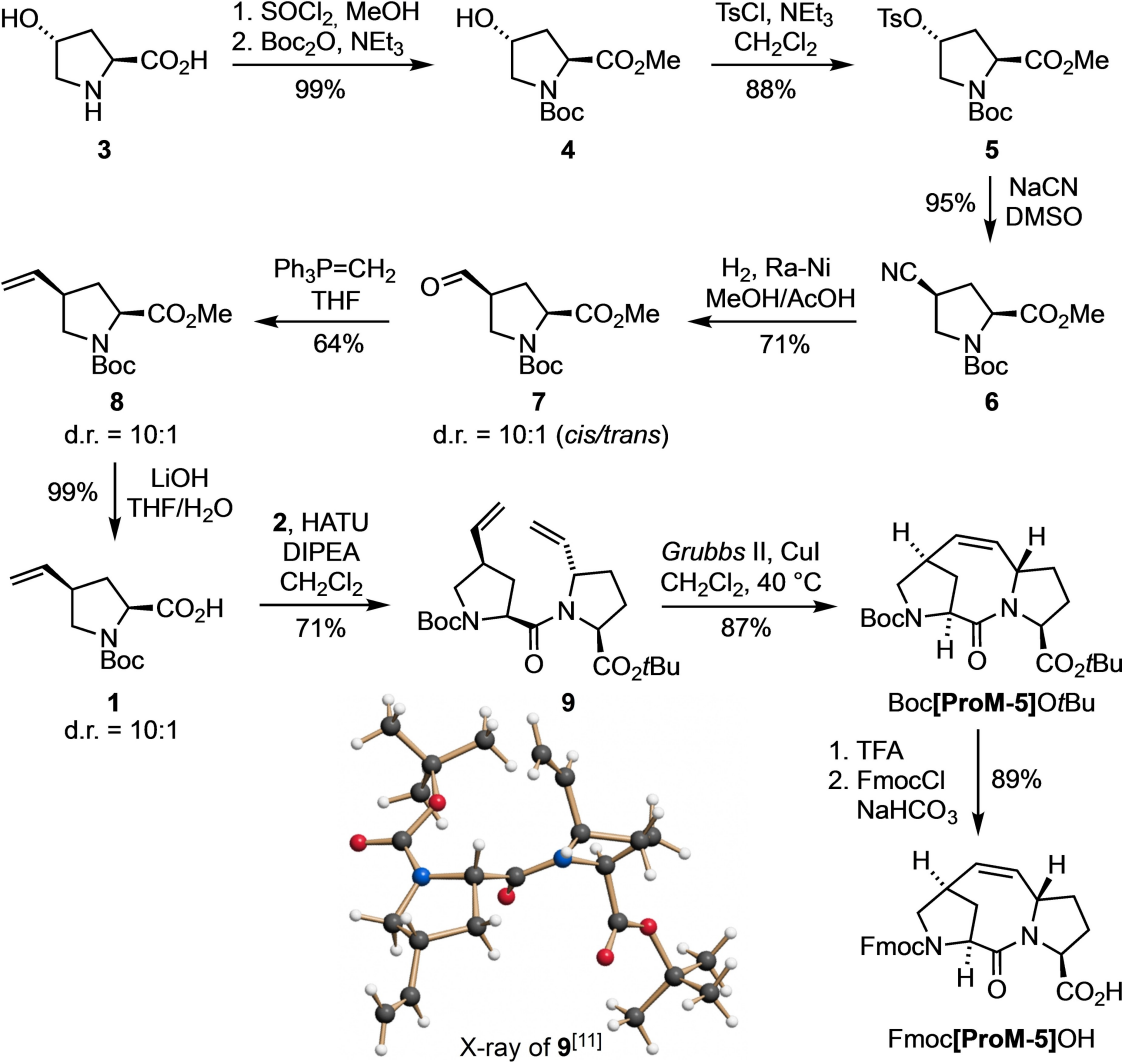
Improved synthesis of building block **1** and Fmoc[**ProM‐5**]OH.

The *trans*‐5‐vinylproline ester **2**, which is an important building block for other ProMs as well,^[9]^ was synthesized via the established four‐step route starting from (*S*)‐proline.^[10]^ For the *N*‐protected *cis*‐4‐vinylproline **1**, on the other hand, we developed an improved synthesis since the original procedure could not be readily scaled up (Scheme 2). As before, we started from *trans*‐4‐hydroxyproline **3**, which was first converted to tosylate **5** via the doubly protected derivative **4**.^[6a]^ The vinyl substituent was then introduced by S_N_2 cyanation, reduction of the nitrile and *Wittig* olefination. While the use of DIBALH did not allow selective reduction of the nitrile function in **6**, we achieved the transformation in 71 % yield through Raney Ni‐catalyzed hydrogenation. Noteworthy, in pyridine/AcOH at 50 °C, we obtained mainly the undesired *trans*‐epimer of **7** (formed by enolization/epimerization of the formyl group). However, at room temperature in an AcOH/MeOH/H_2_O solvent mixture the hydrogenation proceeded under retention of configuration to give mainly the *cis*‐aldehyde **7** (d. r.=10 : 1) in good yield even on a multi‐gram scale. *Wittig* methylenation of **7** then afforded the vinyl‐proline derivative **8** from which the building block **1** was obtained by ester hydrolysis in an improved overall yield of 37 % over 6 steps.

Without separation, the 10 : 1 (*cis*/*trans*) mixture of carboxylic acid **1** was then coupled with the amine **2** using HATU as a reagent to yield the pure dipeptide **9** in an improved yield of 71 % (on a gram scale) after separating off the minor diastereomer. The configuration of **9** was confirmed by X‐ray crystal structure analysis.^[11]^ Treatment of **9** with 20 mol% of the *Grubbs* II catalyst in the presence of 30 mol% of copper iodide as a phosphine scavenger^[12]^ afforded the tricyclic compound Boc[**ProM‐5**]O*t*Bu, again in an improved yield (87 %). Finally, double deprotection (TFA) and treatment with Fmoc‐Cl afforded the desired *N*‐protected acid Fmoc[**ProM‐5**]OH (55 % overall yield from **1** and **2** over 3 steps), which was now ready to be used in solid phase peptide synthesis.

With sufficient amounts of the α‐helix‐inducing *N*‐cap in our hands, we next turned our attention to the synthesis of peptides as potential inhibitors of the ACE2 spike‐RBD interaction.^[5]^ For this, we selected the relevant region of the in total 30 amino acids containing α1 motif as a lead and investigated three main series of peptides (P‐1 to P‐3, see Table 1). In all cases three variants were prepared, one containing the **βHAsp‐ProM‐5** *N‐*cap, one reference with two prolines instead of the **ProM‐5** unit, and as a second reference the *N*‐acetylated parent peptide without any special *N*‐cap. The synthesis of the peptides was performed using automated solid‐phase peptide synthesis (SPPS) on Rink amide resin. Only the non‐natural βHAsp‐ and **ProM‐5**‐containing *N*‐caps were coupled manually under minimization of reagent excess. All peptides were obtained in high purity after HPLC and their identity was confirmed by LC–MS (see Supporting Information, Table S1).

**Table 1:** Overview of the synthesized peptides and their theoretically predicted fractional helicities *f_H_* (pred), measured fractional helicities *via* CD spectroscopy *f_H_* (exp) as well as dissociation constants K_D_ determined from binding affinity measurements on SARS‐CoV‐2 spike‐RBD. N‐Caps are highlighted in blue or red, additional or exchanged amino acid residues are highlighted in green, dark red, violet or yellow, respectively.

#	Peptide sequence	*f_H_* (pred)^[a]^	*f_H_* (exp)^[b]^	K_D_ [μM]^[c]^
P‐1‐1	Ac‐**βHAsp‐[ProM‐5]**‐QAKTFLDKFNHEAEDLFYQ‐NH_2_	–	4 %	–
P‐1‐2	Ac‐**βHAsp**‐**P**‐**P**‐QAKTFLDKFNHEAEDLFYQ‐NH_2_	0.8 %	4 %	–
P‐1‐3	Ac‐QAKTFLDKFNHEAEDLFYQ‐NH_2_	1.2 %	≤1 %	–
P‐2‐1	Ac‐**βHAsp‐[ProM‐5]**‐ **E** QAKTFLDKFNHEAEDLFYQ **K** ‐NH_2_	–	15 %	1.21±0.36
P‐2‐2	Ac‐**βHAsp**‐**P**‐**P**‐ **E** QAKTFLDKFNHEAEDLFYQ **K** ‐NH_2_	1.1 %	4 %	**0.062±0.017**
P‐2‐3	Ac‐ **E** QAKTFLDKFNHEAEDLFYQ **K** ‐NH_2_	1.6 %	5 %	–
P‐3‐1	Ac‐**βHAsp‐[ProM‐5]**‐ **E** QAK **AAA** DK **AA** HEAE **AAA** YQ **K** ‐NH_2_	–	63 %	13.6±6.1
P‐3‐2	Ac‐**βHAsp**‐**P**‐**P**‐ **E** QAK **AAA** DK **AA** HEAE **AAA** YQ **K** ‐NH_2_	26 %	41 %	**0.77±0.12**
P‐3‐3	Ac‐ **E** QAK **AAA** DK **AA** HEAE **AAA** YQ **K** ‐NH_2_	40 %	42 %	–
P‐4	Ac‐**βHAsp**‐**P**‐**P**‐ **AQHAAEAAAEQEYAKADAKKA** ‐NH_2_	6 %	5 %	36.5±26.3
P‐5	Ac‐**βHAsp**‐**P**‐**P**‐ **E** QAK **AAA** DK **AA** HE‐NH_2_	10 %	2 %	19.0±9.8
P‐6	Ac‐**βHAsp**‐**P**‐**P**‐ **E** QAK **AAA** DK **AA** HEAE **AAA** YQ **AAL** ‐NH_2_	30 %	40 %	24.8±7.8

[a] Helix content values predicted by the algorithm AGADIR at 277 K and pH=7. [b] Calculated from the experimental CD spectra using the CDSSTR algorithm on DichroWeb. [c] Determined via microscale thermophoresis (MST) on receptor binding domain (RBD) from wildtype SARS‐CoV‐2 (2019‐nCoV) in capillaries free in solution. No values are given for cases where the signal‐to‐noise ratio did not reach the required threshold to consider this a binding event.

To experimentally determine the α‐helicity, i. e. the fraction of peptide adopting an α‐helical conformation, we used circular dichroism (CD) spectroscopy. The fractional helicities (*f_H_*) were then calculated from the spectra using the DichroWeb online analysis tool with the CDSSTR algorithm.^[13]^

**Figure 2:**
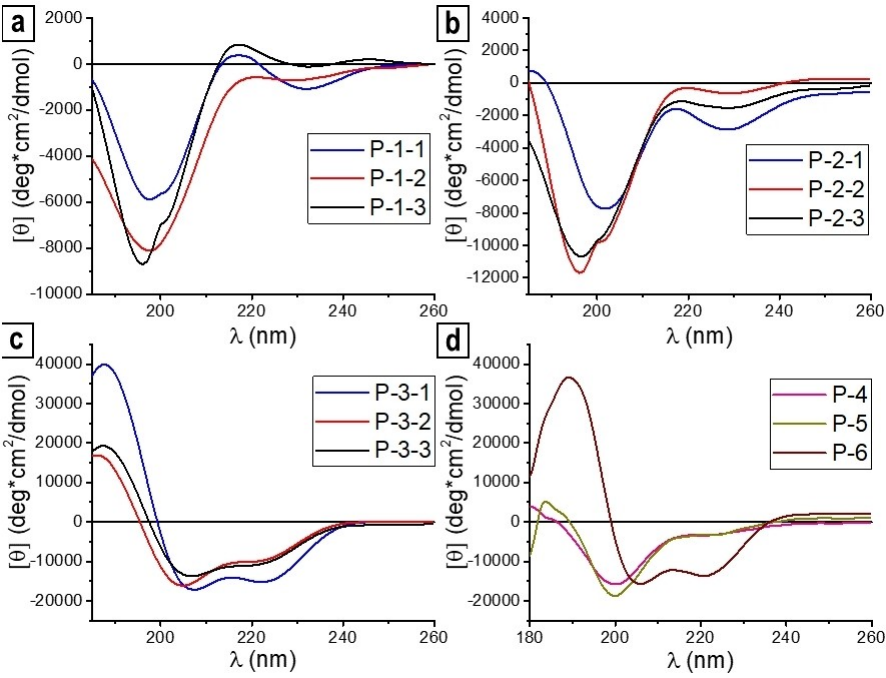
CD spectra of all synthesized peptides listed in Table 1 at concentrations of 60 μM in 10 mM sodium phosphate buffer at pH=7.4 and 4 °C, revealing a pronounced α‐helicity of P‐3‐1, P‐3‐2, P‐3‐3 and P‐6.

For the first series of peptides (P‐1‐1 to P‐1‐3), we chose the nineteen amino acid sequence of α1 from Gln24 to Gln42.^[5]^ However, none of the resulting peptides showed any noteworthy tendency to form an α‐helix, independent of which *N‐*cap was used (Table 1, entries 1–3). This is directly reflected in the corresponding CD spectra (Figure 2a), where the characteristic curve for an α‐helix would show negative maxima at 222 nm and 208 nm and a positive maximum at 193 nm,^[14]^ which is clearly not the case here. Only a slightly higher α‐helicity of 4 % was found for the *N‐*capped peptides P‐1‐1 and P‐1‐2 as compared to the reference peptide P‐1‐3. Therefore, we extended the sequence by also including Glu23 of α1 at the *N‐*terminal side to increase the space between the *N*‐cap and the binding amino acids and to possibly stabilize the α‐helix. Noteworthy, glutamic acid (E) is frequently found in α‐helices^[15]^ and was also used in our previously described α‐helical peptides in the position next to the **ProM‐5** *N‐*cap.^[6a]^

Additionally, we added a *C*‐terminal lysine (K), since positively charged amino acids at the *C*‐terminus are also known to stabilize α‐helices.^[16]^ These considerations were backed by calculations of the α‐helicity using the online tool AGADIR,^[17]^ which allows to predict the fractional helicity of peptides with a given primary structure (Supporting Information, Table S1). While at first glance this second series of peptides (P‐2‐1 to P‐2‐3) showed no significant change in the CD spectra (Figure 2b), the DichroWeb analysis, however, revealed a significantly increased helical content of 15 % for the **ProM‐5** containing peptide (P‐2‐1). In contrast, the reference peptides P‐2‐2 and P‐2‐3 displayed only low α‐helicities of 4 % and 5 %, respectively.

To further increase the helical content, while maintaining the amino acids relevant for binding to spike‐RBD, the sequence was modified by replacing innocent amino acids by alanine, which has the highest α‐helix stabilizing effect.^[18]^ As “binding” amino acids known to face spike‐RBD^[5]^ (Figure 1a), Gln24, D30, Lys31, His33, Glu37, Tyr41 and Gln42 were conserved. Using the AGADIR software tool again, the effect of a stepwise replacement of the other amino acids by alanine on the fractional helicities of the resulting peptides was predicted (Supporting Information, Table S1). In this way, an *in silico* optimized sequence (P‐3‐3) was identified with a predicted helicity of 40 %. The corresponding peptides (P‐3‐1 to P‐3‐3) were then synthesized and analyzed for their helicities by CD spectroscopy (Figure 2c). Noteworthy, this enrichment of the parent sequence with the hydrophobic amino acid alanine did not lead to an observable change in the (very high) aqueous solubility of the resulting peptides.

Much to our satisfaction, the experimentally determined helicity (42 %) of the parent (“non‐*N‐*capped”, i. e. *N*‐acetylated) peptide P‐3‐3 corresponded closely to the predicted value. Moreover, the **βHAsp‐ProM‐5**‐containing peptide (P‐3‐1) showed an even stronger degree of helicity (63 %). Interestingly, even the diproline‐capped peptide (P‐3‐2) displayed an α‐helical content of 41 % which is higher than the predicted value of 26 %. This discrepancy may be due to proline acting as a “helix breaker” in the predictions^[19]^ and/or to a specific role of βHAsp.^[17]^ In any case, the fact that there is almost no difference between the two reference peptides, P‐3‐2 and P‐3‐3, suggests that the *N*‐terminal prolines have no significant effect on the helical content. However, the much higher helicity (63 %) of the **ProM‐5** capped peptide (P‐3‐1) in comparison to the reference peptides must be considered as a proof of the helix‐inducing effect of our synthetic *N‐*cap.

**Figure 3:**
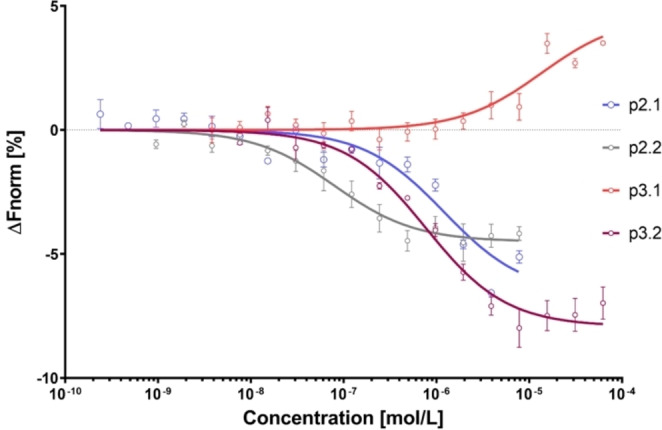
MST binding curves from experiments using different concentrations of ACE2‐derived peptides p2.1, p2.2, p3.1, and p3.2 against a constant concentration of fluorescently labelled RBD. Error bars: SEM with N≥3.

The binding affinity of all synthesized peptides towards the receptor binding domain (RBD) of Spike from wildtype SARS‐CoV‐2 (2019‐nCoV) was determined using microscale thermophoresis (MST), and the obtained dissociation constants (K_D_) were set in relation to the fractional helicities of the corresponding peptides (Table 1). No significant binding was observed for the non‐helical peptide series P‐1‐1 to P‐1‐3. While the reference peptide P‐2‐3 also showed no binding, the **βHAsp‐ProM‐5**‐capped peptide P‐2‐1, which is the only one of the P‐2 series to exhibit a significant degree of helicity, was found to bind to spike with a K_D_ of 1.2 μM (see Figure 3). This shows that our synthetic *N*‐cap can indeed not only be used to induce helicities but also to improve binding affinities in certain peptides.

Remarkably, we found that the corresponding **Ac‐βHAsp‐P‐P** capped peptide P‐2‐2 binds with an even much stronger affinity of 62 nM. This result suggests that an increased helicity does not necessarily lead to an improved binding affinity, and that the helical content of the peptides appears to not directly correlate to the measured binding. The data obtained for the alanine‐enriched peptides P‐3‐1 to P‐3‐3 seems to further underline this behavior. In this series, the **βHAsp‐ProM‐5**‐capped peptide P‐3‐1, which exhibits the highest helical content, shows only a K_D_ of 13 μM, while the **Ac‐βHAsp‐P‐P**‐capped peptide P‐3‐2 binds at nanomolar concentrations (770 nM) despite its lower helicity. Again, the uncapped analogue P‐3‐3 (control) showed no significant binding to the RBD of the spike protein.

To shed some more light on these surprising results, we synthesized and studied three additional peptides related to P‐3‐2 (as one of the nanomolar binding compounds), all containing an **Ac‐βHAsp‐P‐P** unit at the *N*‐terminus. Peptide P‐4, which contains the same amino acids as P‐3‐2 but in randomized order, only showed a very weak binding (36 μM). Peptide P‐5, which is *C*‐terminally shortened by eight amino acid units (to Glu35), displayed an affinity of 19 μM, which may indicate some involvement of the **Ac‐βHAsp‐P‐P** unit in the binding process. In contrast, the elongated peptide P‐6, which shows higher helicity and bears a leucine residue at the *C*‐terminus (AAL instead of K) that could participate in the binding to the spike protein,^[5]^ exhibited only a low affinity of 24 μM.

Taken together, these results suggest that the comparably strong binding affinities of the **Ac‐βHAsp‐P‐P** capped peptides P‐2‐2 and P‐3‐2 result from a lucky but subtle interplay of different effects involving α‐helical content, a favorable presentation of relevant amino acid side chains to the partner protein and, possibly, a direct contribution of the *N*‐cap to the binding event. While a certain degree of helicity appears to be beneficial for an α1‐derived peptide to undergo binding, a too pronounced helicity (as determined for P‐3‐1) may be detrimental in this context. The binding site of spike‐RBD to ACE2 is most likely an allosteric site undergoing an induced fit upon interacting with ACE2.^[20]^ Consequently, an α‐helix that is too stiff might be problematic in the formation of a stable RBD‐peptide complex. Further amino acids within α‐1 or other protein epitopes of ACE2 might also be relevant for binding.

An additional comparison of helical wheel analyses^[21]^ for the **Ac‐βHAsp‐P‐P** *N*‐capped peptides P‐2‐2 and P‐3‐2 with their respective references (P‐2‐3 and P‐3‐3) shows that the introduction of the *N*‐cap results in a more profound arrangement of the polar binding residues on one face of the helix (Supporting Information, Figure S27). The Ac‐βHAsp part of the *N‐*cap may have some additional enthalpic contribution in binding due to an additional negative charge. The higher flexibility of the diproline unit may allow for a beneficial placement of this modified N‐terminus in contrast to the stiffer **ProM‐5** unit, which may explain the difference in binding between P‐2‐1 and P‐2‐2, as well as P‐3‐1 and P‐3‐2, respectively. While the exact influence of the **Ac‐βHAsp‐P‐P** unit remains unknown, P‐2‐2 serves as an impressive example of a modified peptide with a very strong binding affinity, which relies only on readily available amino acid building blocks and no complex synthetic moieties or cross‐linkages.

## Conclusion

In the search for inhibitors of the ACE2 SARS‐CoV‐2 protein‐protein interaction as potential anti‐COVID‐19 agents, we have synthesized and characterized a series of peptides mimicking the relevant region of the α1‐helix of ACE2. To induce α‐helix nucleation in the peptides, we initially exploited the conformationally restricted diproline analogue **ProM‐5** as an *N*‐cap, for which an improved synthesis was elaborated. As a first success, the **ProM‐5**‐containing peptide P‐2‐1 was found to bind to the target protein with a respectable K_D_ of 1.2 μM. As a second method for increasing the α‐helical content of the peptides, we replaced 8 (out of 21) non‐binding amino acids of the core sequence by alanine, resulting in the alanine‐enriched **Ac‐βHAsp‐ProM‐5**‐capped peptide (P‐3‐1), which showed a weaker affinity (12 μM) despite its pronounced helicity of 63 %. However, it is particularly noteworthy that the corresponding peptides P‐2‐2 and P‐3‐2 (which were originally intended as controls in which the ProM‐5 unit was replaced by two prolines) showed even better binding to the spike protein (K_D_=62 nM and 770 nM, respectively).

In any case, our study has resulted in the identification of the comparably short α1‐helix mimicking peptide P‐2‐2 (comprising only 24  amino acids) which binds to the spike RBD with excellent (nanomolar) affinity. Additionally, we demonstrated that α‐helical preorganization only partly contributes to the binding affinity. We are confident that our results open some interesting insights which may contribute to the development of even more powerful spike RBD binding peptides as potential COVID‐19 drugs in the future.

## Experimental Section

**Fmoc‐ProM‐5‐OH** ^[6a]^ was synthesized as detailed in the Supporting Information. Peptides were prepared by Solid Phase Peptide Synthesis using a MultiSynTech Syro automated peptide synthesizer employing 30 mg batches of Rink‐amide resin (Merck) with a NovaPEG linker (surface conc.=0.48 mmol/g). All coupling steps were performed in DMF using equimolar amounts (8.00 eq.) of Fmoc‐protected amino acids, DIC and oxyma. Side chain functional groups were protected with acid labile protecting groups. At the end of each coupling cycle the Fmoc protecting group was cleaved with 30 % piperidine in DMF. *N*‐caps (i. e. Fmoc‐**ProM‐5**‐OH, Fmoc‐L‐Pro‐OH or Fmoc‐L‐βHAsp‐*t*Bu‐OH) were manually coupled to the synthesized (still resin‐attached) peptides using the Fmoc‐protected amino acid (2.0 eq.), HATU (2.0 eq.) and DIPE (2.0 eq.) in 300 μL DMF/CH_2_Cl_2_ (9 : 1). After shaking at room temp for 2 hours, the resin was washed with DMF, CH_2_Cl_2_, MeOH and Et_2_O and dried. After completing the peptide synthesis, the free *N*‐terminus was acetylated by treatment with of 20 eq. of Ac_2_O and 20 eq. of DIPEA in 300 μL of CH_2_Cl_2_ for 30 min at room temp and then washing with DMF, CH_2_Cl_2_, MeOH and Et_2_O. Finally, the peptides were cleaved off the resin by treatment with 1 ml of a mixture of TFA, triisopropylsilane and water (95 : 2.5 : 2.5) and shaking for 3 h at room temp. After filtration, the resin was washed with 0.2 mL of TFA and the combined filtrates were added to 10 ml of cold Et_2_O and stored at −20 °C for 16 h. The precipitated peptides were isolated by centrifugation, washed several times with cold Et_2_O, dissolved in *t*BuOH/water (1 : 4) and lyophilized. Peptides were purified by preparative RP‐HPLC (Hitachi Elite LaChrom system with a Macherey‐Nagel VP 250/8 Nucleodur 100–5 C18ec column) using 0.1 % aqueous TFA and acetonitrile as solvents (linear gradient of 30 % to 60 % acetonitrile over 30 min; flow rate=1.5 ml/min). The solvent was removed from all relevant fractions using a Horizon Technology Xcel Vap in an air flow gradient from 880 to 1640 mbar in 20 minutes at 65 °C prior to lyophilization. The product identity was confirmed by LC‐ESI‐MS analysis using a Merck Chromolith Performance RP‐18e end‐capped 100–4.6 mm HPLC column coupled to a ThermoScientific LTQ‐XL linear ion trap mass spectrometer (gradient: 20 % to 70 % acetonitrile in 0.1 % aqueous formic acid over 15 minutes). Purities were determined by integration of peaks in the UV chromatogram. CD spectra were recorded on a JASCO J715 Spectropolarimeter at wavelengths from 180 to 260 nm in steps of 0.2 nm using 60 μM solution of the peptides in 10 mM phosphate buffer (pH=7.4). Binding affinities were determined by Microscale Thermophoresis (MST) at 22 °C on a Monolith NT.115 Pico instrument (NanoTemper Technologies) with an excitation power of 20 % and a MST power of 40 % using His‐tagged wildtype RBD from SARS‐CoV‐2 Spike protein.

## Conflict of interest

The authors declare no conflict of interest.

1

## Supporting information

As a service to our authors and readers, this journal provides supporting information supplied by the authors. Such materials are peer reviewed and may be re‐organized for online delivery, but are not copy‐edited or typeset. Technical support issues arising from supporting information (other than missing files) should be addressed to the authors.

Supporting Information

## Data Availability

The data that support the findings of this study are available in the supplementary material of this article.
