# Supplementary material for: Synthetic α‐Helical Peptides as Potential Inhibitors of the ACE2 SARS‐CoV‐2 Interaction
Source: Chembiochem. 2022 Jul 14;23(17):e202200372. doi: 10.1002/cbic.202200372 (PMC9350387; doi:10.1002/cbic.202200372)
Supplement: Supplementary file 1 — Supporting Information [file CBIC-23-0-s001.pdf]

ChemBioChem

Supporting Information

Synthetic α-Helical Peptides as Potential Inhibitors of the ACE2 SARS-CoV-2 Interaction

Pascal M. Engelhardt, Sebastián Florez-Rueda, Marco Drexelius, Jörg-Martin Neudörfl, Daniel Lauster, Christian P. R. Hackenberger, Ronald Kühne, Ines Neundorf,* and Hans-Günther Schmalz*

1 Chemical Synthesis

1.1 General experimental methods

Moisture or air sensitive substances were handled in flame-dried glassware under inert atmosphere using an oil pump vacuum/argon double manifold. As protective gas *Argon BIP (Air Products)* with a purity of 99.9997% was used. The addition of solids was performed under argon counterflow, liquids and solutions were added *via* plastic syringes with steel cannulas which were pre-dried at 80 °C. Reactions at low temperature were conducted by means of ice/water mixtures for 0 °C and by means of dry ice/*iso*-propanol mixtures for as low as -78 °C.

The solvents and reagents used for syntheses were obtained from *Acros Organics, AK Scientific, Alfa Aesar, Cambridge Isotope Labs, Eastman Organic Chemicals, Fisher Chemical, TCI, Sigma-Aldrich* and *Strem Chemicals* with purities of ≥97%. Dry THF was produced from HPLC grade THF that was dried over sodium/benzophenone and freshly distilled under argon atmosphere. Dry CH_2Cl_2 (HPLC grade) was dried over CaH_2 and freshly distilled under argon atmosphere. Moisture sensitive catalysts were stored in a *mBraun LABmaster 130* glovebox under argon atmosphere at < 1 ppm O_2 and < 1 ppm H_2O.

Thin layer chromatography (TLC) was carried out using *Merck* TLC Silica Gel 60 F_{254} plates with a thickness of 0.25 mm. The separated substances were visualized either under UV-light (254 nm) or using a potassium permanganate staining solution (1.5 g $KMnO_4$, 10 g K_2CO_3 and 1.25 ml 10% NaOH in 200 ml water). For column chromatography *Acros silica gel* 60 A (0.060-0.200 mm) was used. The flow rate of the eluent was accelerated by excess pressure. Ratios of solvents used are given in volume per volume.

Gas chromatograms were recorded using an *Agilent* HP6890 instrument coupled to a *MSD* 5937 N mass detector (GC-MS). As capillary column an Optima-1-MS (30 m x 0.25 mm Ø) from *Macherey-Nagel* was used. Hydrogen served as the carrier gas (temperature program 50 – 300 °C, 17 min). The stated signals refer to the ratio m/z, the intensities are given relative to the highest signal (100%).

NMR spectra were recorded on *Bruker* DPX300 (^{1}H: 300 MHz, ^{13}C: 75 MHz) and Avance-II 500 (^{1}H: 500 MHz, ^{13}C: 125 MHz) spectrometer at 298 K. Chemical shifts δ are given in parts per million (ppm) in reference to the signal of tetramethylsilane (TMS) [δ (^{1}H NMR) = 0.00 ppm]. As solvent deuterated chloroform $CDCl_3$ [δ (^{1}H) = 7.26 ppm; δ (^{13}C) = 77.16 ppm] was used. The multiplicities of signals in ^{1}H spectra are abbreviated as s = singlet, d = doublet, t = triplet, q = quartet, dd = doublet of doublets, bs = broad singlet, and m = multiplet. ^{13}C Spectra were recorded as ^{1}H-decoupled and partly as APT (Attached Proton Test) spectra. Signal assignements were supported by two-dimensional NMR spectra, (*H,H*-COSY, HMBC and HMQC).

High resolution (HR-MS) mass spectra were recorded using electrospray iionization (ESI) on a *THERMO Scientific* LTQ Orbitrap XL spectrometer and the signals were compared to calculated values (Errors < 1 ppm).

Infrared (IR) spectra were recorded on a *Perkin Elmer* Spectrum Two FT-IR spectrometer with ATR technology. The positions of absorption bands are given in wavenumbers (cm^{-1}), their intensities are stated and abbreviated as s = strong, m = medium, w = weak and b = broad.

The specific rotation of optically active substances was measured on an *Anton Paar* MCP200 polarimeter at the individually stated wavelengths at 20 °C in dry chloroform. The concentrations of the measured solutions are stated in g/100 ml.

Melting points were determined on a *Wepa* apotec melting point measurement instrument.

1.2 Synthetic procedures and analytical data

(2*S*,4*R*)-1-(*tert*-butyl)-2-methyl-4-hydroxypyrrolidine-1,2-dicarboxylate (4)

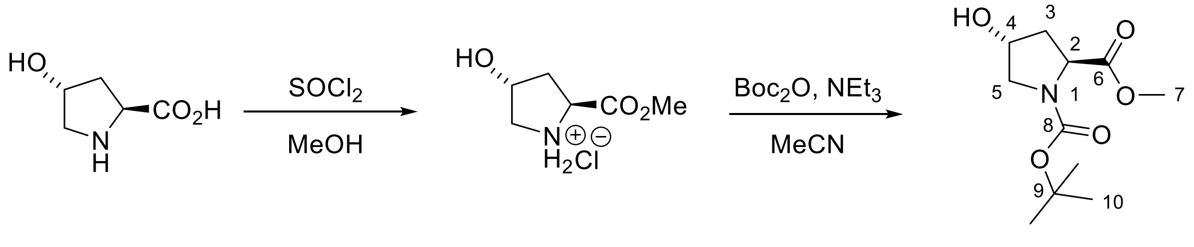

3 **4**

According to a procedure by *V. Hack et al.*,[1] a solution of 14.5 g (111 mmol, 1.00 eq.) (*S*)-*trans*-4-hydroxyprolin (**3**) in 110 ml methanol was cooled to 0 °C and 12.0 ml (166 mmol, 1.50 eq.) thionyl chloride were added dropwise. The solution was stirred at room temperature for 16 hours, after which the solvent was evaporated under reduced pressure. The resulting hydrochloride was suspended in 145 ml acetonitrile and cooled to 0 °C. 31.0 ml (222 mmol, 2.00 eq.) triethylamine and 26.6 g (122 mmol, 1.10 eq.) Boc_2O were added, and the mixture was stirred at room temperature for 22 hours. After addition of 450 ml CH_2Cl_2, the layers were separated and the organic layer was washed three times with each 300 ml of 1.0 M aqueous $KHSO_4$ solution, dried over $MgSO_4$ and concentrated *in vacuo* to afford 26.9 g (110 mmol, 99%, Lit.[1]: 98%) of product **4** as a colorless oil. **R_f** = 0.19 (SiO_2, EtOAc/cHex 1:2); **$[\alpha]_D^{20}$** = -64.22 ° (c = 2.185 g/100 cm^3, $CHCl_3$); **^{1}H NMR:** (300 MHz, $CDCl_3$, mixture of rotamers) δ [ppm] = 4.47 – 4.32 (m, 2H, H-2, H-4), 3.70 (s, 3H, H-7), 3.62 – 3.39 (m, 2H, H-5), 2.95 (s, 1H, OH), 2.33 – 2.19 (m, 1H, H-3), 2.07 – 1.95 (m, 1H, H-3'), 1.42/1.37 (2 x s, 9H, H-10); **^{13}C NMR:** (75 MHz, $CDCl_3$, mixture of rotamers) δ [ppm] = 173.8 (C-6), 154.1 (C-8), 80.6 (C-9), 70.1/69.4 (C-4), 58.2/57.6 (C-2), 54.8 (C-5), 52.3/52.1 (C-7), 39.2/38.5 (C-3), 28.5/28.3 (C-10); **IR:** (ATR) ṽ [cm^{-1}] = 3427 (b), 2978 (m), 2951 (w), 2886 (w), 1747 (m), 1674 (s), 1478 (w), 1399 (s), 1366 (m), 1341 (w), 1278 (w), 1257 (w), 1202 (m), 1156 (s), 1125 (m), 1086 (m), 1056 (w), 1029 (w), 981 (w), 959 (w), 916 (w), 895 (m), 855 (w), 773 (m), 752 (w), 552 (w), 493 (w), 463 (w); **MS:** (DIP-EI, 70 eV) m/z (%) = 245 $[M]^+$ (1), 186 $[M\text{-}CO_2Me]^+$ (25), 144 $[M\text{-}Boc]^+$ (31), 130 (77), 86 (100), 68 (27), 57 (99), 41 (75).

1-(*tert*-Butyl)-2-methyl-(2*S*,4*R*)-4-(tosyloxy)pyrrolidine-1,2-dicarboxylate (5)

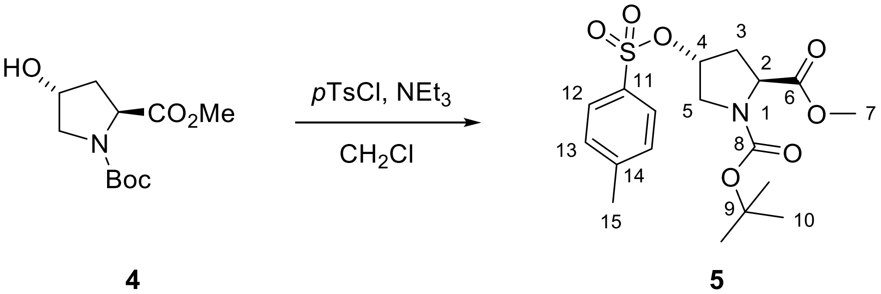

4 **5**

According to a procedure by *V. Hack et al.*,[1] to a solution of 25.5 g (104 mmol, 1.00 eq.) hydroxyproline **4** in 170 ml CH_2Cl_2 were added 43.5 ml (312 mmol, 3.0 eq.) triethylamine and the mixture was cooled to 0 °C. After addition of 43.6 g (229 mmol, 2.20 eq.) para-toluenesulfony chloride the mixture was stirred at room temperature for 20 hours. Once the reaction was determined complete *via* TLC, 120 ml sat. aqueous $NaHCO_3$ were added, the layers were separated, and the aqueous layer extracted three times with each 120 ml CH_2Cl_2. The combined organic layers were dried over $MgSO_4$, the solvent evaporated under reduced pressure and the residue was purified by column chromatography (SiO_2, EtOAc/cHex 1:4) to afford 36.4 g (91.1 mmol, 88%, Lit.[1]: 88%) of product **5** as a yellow solid. **R_f** = 0.35 (SiO_2, EtOAc/cHex 1:2); **m.p.** = 74.4 °C; **$[\alpha]_D^{20}$** = -32.72 ° (c = 1.835 g/100 cm^3, $CHCl_3$); **^{1}H NMR:** (300 MHz, $CDCl_3$, mixture of rotamers) δ [ppm] = 7.75 (d, $^3J_{HH}$ = 8.2 Hz, 2H, H-12), 7.33 (d, $^3J_{HH}$ = 8.1 Hz, 2H, H-13), 5.07 – 4.96 (m, 1H, H-4), 4.39 – 4.24 (m, 1H, H-2), 3.69 (s, 3H, H-7), 3.63 – 3.51 (m, 2H, H-5), 2.59 – 2.35 (m, 1H, H-3), 2.43 (s, 3H, H-15), 2.17 – 2.08 (m, 1H, H-3'), 1.39/1.36 (2 x s, 9H, H-10); **^{13}C NMR:** (75 MHz, $CDCl_3$, mixture of rotamers) δ [ppm] = 172.8 (C-6), 153.2 (C-8), 145.4 (C-14), 133.4 (C-11), 130.2 (C-13), 127.8 (C-12), 80.8 (C-9), 79.1/78.5 (C-4), 57.5/57.1 (C-2), 52.3/52.2 (C-7), 52.2/51.9 (C-5), 37.3/36.1 (C-3), 28.3/28.3 (C-10), 21.7 (C-15); **IR:** (ATR) ṽ [cm^{-1}] = 2982 (m), 2955 (w),

2939 (w), 2898 (w), 1749 (s), 1704 (s), 1597 (w), 1475 (w), 1455 (w), 1439 (w), 1401 (s), 1359 (s), 1343 (m), 1312 (w), 1280 (w), 1260 (w), 1214 (m), 1176 (s), 1154 (s), 1126 /m), 1098 (m), 1056 (w), 1020 (w), 1010 (w), 972 (w), 952 (m), 907 (s), 874 (w), 854 (m), 819 (m), 778 (w), 759 (w), 745 (w), 657 (m), 633 (w), 569 (m), 555 (s), 502 (w), 491 (m); **MS:** (DIP-EI, 70 eV) m/z (%) = 399 $[M]^+$ (2), 341 (3), 327 (5), 281 (14), 207 (20), 168 (14), 147 (12), 126 (17), 112 (67), 73 (38), 68 (89), 57 (100).

1-(*tert*-butyl)-2-methyl-(2*S*,4*S*)-4-cyanopyrrolidine-1,2-dicarboxylate (6)

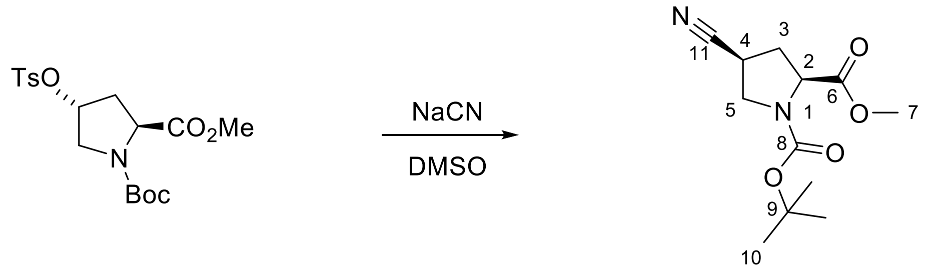

5 **6**

According to a modified procedure by *V. Hack et al.*,[1] to a solution of 11.9 g (25.0 mmol, 1.00 eq.) tosylate **5** in 36 ml anhydrous DMSO under inert atmosphere were added 4.38 g (77.6 mmol, 3.10 eq.) previously dried NaCN. The solution was heated to 55 °C and stirred for 72 hours, after which reaction control *via* NMR showed full conversion of the starting material. After addition of 20 ml of brine and 20 ml of water the mixture was extracted five times with each 50 ml MTBE. The combined organic layers were dried over $MgSO_4$ and the solvent evaporated under reduced pressure to afford 3.67 g (14.4 mmol, 95%, Lit.[1]: 94%) of crude product **6** as a colorless solid, which was used in the next step without further purification. **R_f** = 0.31 (SiO_2, EtOAc/cHex 1:2); **m.p.** = 67.1 °C; **$[\alpha]_D^{20}$** = -30.73 ° (c = 0.665 g/100 cm^3, $CHCl_3$); **^{1}H NMR:** (300 MHz, $CDCl_3$, mixture of rotamers) δ [ppm] = 4.47 – 4.24 (m, 1H, H-2), 3.99 – 3.84 (m, 1H, H-5), 3.75/3.72 (2 x s, 3H, H-7), 3.68 – 3.62 (m, 1H, H-5'), 3.26 – 3.03 (m, 1H, H-4), 2.73 – 2.59 (m, 1H, H-3), 2.36 – 2.21 (m, 1H, H-3'), 1.44/1.38 (2 x s, 9H, H-10); **^{13}C NMR:** (75 MHz, $CDCl_3$, mixture of rotamers) δ [ppm] = 171.7 (C-6), 153.0 (C-8), 118.9 (C-11), 81.2 (C-9), 58.2/57.9 (C-2), 52.6/52.5 (C-7), 49.3/49.1 (C-5), 34.5/33.5 (C-3), 28.3/28.2 (C-10), 27.3/26.6 (C-4); **IR:** (ATR) ṽ [cm^{-1}] = 2977 (m), 2901 (w), 2248 (w), 1748 (m), 1698 (s), 1478 (w), 1454 (w), 1437 (w), 1394 (s), 1367 (m), 1281 (w), 1259 (m), 1205 (m), 1158 (s), 1122 (m), 1066 (m), 990 (w), 896 (w), 865 (w), 771 (w), 586 (w), 555 (w); **MS:** (DIP-EI, 70 eV) m/z (%) = 253 $[M-H]^+$ (1), 195 $[M-CO_2Me]^+$ (12), 153 $[M-Boc]^+$ (21), 139 (5), 95 (48), 68 (8), 57 (100).

1-(*tert*-butyl)-2-methyl-(2*S*,4*S*)-4-formylpyrrolidine-1,2-dicarboxylate (7)

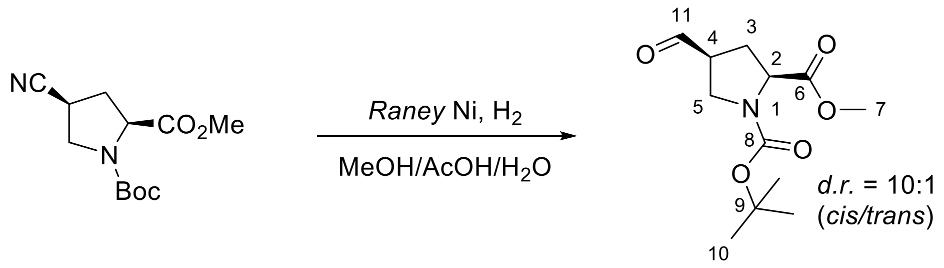

6 **7**

To a solution of 4.90 g (19.3 mmol, 1.0 eq.) nitrile **6** in 39 ml of a 1:2 mixture of methanol and 50% aqueous acetic acid were added 11.3 g (96.3 mmol, 5.0 eq.) of a 50% suspension of *Raney* nickel in water. The reaction flask was flooded with hydrogen gas and the mixture vigorously stirred at room temperature for a total of 4 hours, while carefully monitoring the progress of the reaction *via* GC-MS. Upon complete conversion the hydrogen gas was removed, and the reaction mixture diluted with 30 ml water. The aqueous layer was extracted three times with each 50 ml ethyl acetate, the combined organic layers were dried over $MgSO_4$, and the solvent evaporated under reduced pressure to obtain 3.53 g (13.7 mmol, 71%) of crude product **7** as a colorless oil as a mixture of isomers (10:1 *cis/trans*; determined *via* GC-MS), which was used in the next step without further purification. **R_f** = 0.21 (SiO_2,

EtOAc/cHex 1:2); **[α]$_D^{20}$** = -26.80 ° (c = 0.245 g/100 cm^3, $CHCl_3$); **^{1}H NMR:** (500 MHz, $CDCl_3$, mixture of rotamers) δ [ppm] = 9.68/9.67 (2 x s, 1H, H-11), 4.40 – 4.27 (m, 1H, H-2), 3.94 – 3.76 (m, 2H, H-5), 3.72/3.69 (2 x s, 3H, H-7) 3.21 – 2.94 (m, 1H, H-4), 2.48 – 2.39 (m, 2H, H-3), 1.47 – 1.39 (m, 9H, H-10); **^{13}C NMR:** (126 MHz, $CDCl_3$, mixture of rotamers) δ [ppm] = 200.6 (C-11), 173.0 (C-6), 153.7 (C-8), 80.7 (C-9), 58.6/58.2 (C-2), 52.4/52.3 (C-7), 49./48.7 (C-4), 46.3 (C-5), 30.9/30.0 (C-3), 28.5/28.4 (C-10); **IR:** (ATR) ṽ [cm^{-1}] = 2978 (m), 2956 (w), 2939 (w), 2889 (w), 1748 (m), 1728 (m), 1697 (s), 1478 (w), 1393 (s), 1366 (s), 1256 (m), 1202 (m), 1159 (s), 1124 (m), 1072 (w), 991 (w), 892 (w), 859 (w), 771 (w), 732 (m), 648 (w), 602 (w), 556 (w); **MS:** (DIP-EI, 70 eV) m/z (%) = 257 [M]$^+$ (1), 198 [M-CO_2Me]$^+$ (10), 156 [M-Boc]$^+$ (19), 142 (24), 126 (7), 98 (58), 80 (6), 68 (34), 57 (100); **HRMS** (ESI): m/z calcd for $C_{12}H_{20}NO_5^+$: 258.1336 [M+H]$^+$; found: 258.1339; m/z calcd for $C_{12}H_{19}NO_5Na^+$: 280.1155 [M+Na]$^+$; found: 280.1159.

1-(*tert*-butyl)-2-methyl-(2*S*,4*R*)-4-vinylpyrrolidine-1,2-dicarboxylate (8)

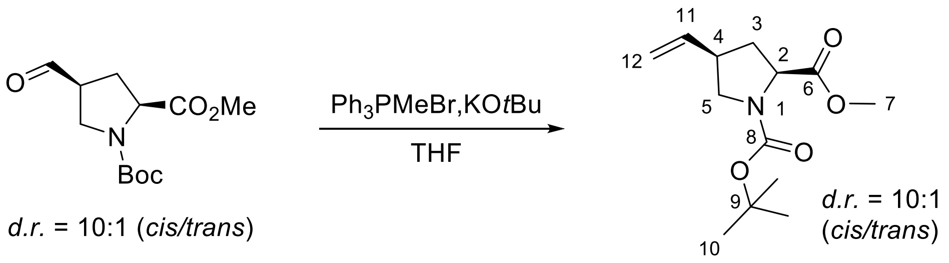

7 **8**

To a suspension of 3.66 g (10.3 mmol, 1.3 eq.) Ph_3PMeBr in 42 ml absolute THF under inert atmosphere at to 0 °C was added 1.06 g (9.47 mmol, 1.2 eq) KO*t*Bu and the mixture was stirred for 30 minutes at 0 °C. A solution of 2.03 g (7.89 mmol, 1.0 eq.) crude aldehyde **7** in 21 ml of dry THF was added dropwise and stirring was continued at room temperature for 2 hours, until full conversion of the starting material was observed *via* TLC. The solvent of the reaction mixture was evaporated under reduced pressure and the residue was directly purified *via* column chromatography (SiO_2, EtOAc/cHex 1:4) to afford 1.28 g (5.01 mmol, 64%) of product **8** as a colorless oil as a mixture of isomers (10:1 *cis/trans*, determined *via* GC-MS). **R$_f$** = 0.31 (SiO_2, EtOAc/cHex 1:2); **[α]$_D^{20}$** = -28.81 ° (c = 0.405 g/100 cm^3, $CHCl_3$); **^{1}H NMR:** (500 MHz, $CDCl_3$, mixture of rotamers) δ [ppm] = 5.71 (m, 1H, H-11), 5.10 (d, $^3J_{HH}$ = 17.1 Hz, 1H, H-12), 5.04 (dd, $^3J_{HH}$ = 10.2 Hz, $^2J_{HH}$ = 4.1 Hz, 1H, H-11'), 4.37 (d, $^3J_{HH}$ = 16.6 Hz, 0.09H, H-2^{trans}), 4.29 – 4.19 (m, 0.91H, H-2^{cis}), 3.78 – 3.66 (m, 1H, H-5), 3.72/3.71 (2x s, 3H, H-7), 3.17 (t, $^3J_{HH}$ = 10.3 Hz, 1H, H-5'^{cis}), 3.09 (t, $^3J_{HH}$ = 9.8 Hz, 0.09H, H5'^{trans}), 2.99 – 2.73 (m, 1H, H-4), 2.43 – 2.38 (m, 0.91H, H-3^{cis}), 2.11 – 2.04 (m, 0.18H, H-3^{trans}), 1.80 – 1.71 (m, 0.91H, H-3'^{cis}), 1.44/1.39 (2 x s, 9H, H-10); **^{13}C NMR:** (126 MHz, $CDCl_3$, mixture of rotamers) δ [ppm] = 173.6/173.4 (C-6), 154.3/153.6 (C-8), 137.6/137.2 (C-11), 116.5/116.2 (C-12), 80.2/80.1 (C-9), 59.4/59.0 (C-2), 52.3/52.1 (C-7), 51.8/51.3 (C-5), 42.7/41.9 (C-4), 37.0/36.2 (C-3), 28.5/28.4 (C-10); **IR:** (ATR) ṽ [cm^{-1}] = 2998 (m), 2956 (w), 2934 (w), 2870 (w), 1751 (m), 1698 (s), 1645 (w), 1479 (w), 1454 (w), 1393 (s), 1365 (m), 1288 (w), 1255 (m), 1198 (m), 1158 (s), 1114 (m), 1073 (w), 1031 (w), 993 (m), 918 (m), 897 (m), 867 (w), 769 (m), 741 (w), 664 (w), 590 (w), 548 (w); **MS:** (DIP-EI, 70 eV) m/z (%) = 255 [M]$^+$ (1), 196 [M-CO_2Me]$^+$ (15), 154 [M-Boc]$^+$ (24), 140 (60), 126 (7), 96 (75), 79 (4), 68 (18), 57 (100); **HRMS** (ESI): m/z calcd for $C_{13}H_{22}NO_4^+$: 256.1543 [M+H]$^+$; found: 256.1547; m/z calcd for $C_{13}H_{21}NO_4Na^+$: 278.1363 [M+Na]$^+$; found: 278.1365.

(2*S*,4*R*)-1-(*tert*-butoxycarbonyl)-4-vinylpyrrolidine-2-carboxylic acid (2)

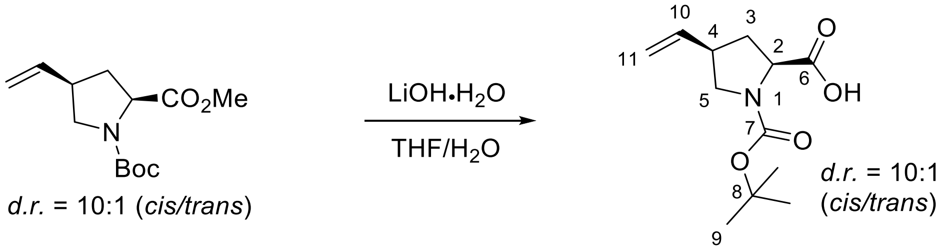

8 **1**

To a solution of 1.04 g (4.07 mmol, 1.0 eq.) methyl ester **8** in 12 ml of a 2:1 mixture of THF and water, 179 mg (4.27 mmol, 1.05 eq.) Lithiumhydroxide monohydrate were added, and the mixture was stirred at room temperature for 18 hours. Once completion of the reaction was determined by TLC, the solution was diluted with 30 ml MTBE then acidified at by addition of 10 ml of 10% aqueous $KHSO_4$ solution to pH = 3. The layers were separated, and the aqueous phase was extracted two times with each 30 ml MTBE. The combined organic layers were dried over $MgSO_4$, and the solvent was evaporated under reduced pressure to afford 976 mg (4.05 mmol, >99%) of product **1** as a colorless solid as a mixture of isomers (10:1 *cis/trans*, determined *via* GC-MS). **R*f*** = 0.24 (SiO_2, EtOAc/cHex/HOAc 1:2:0.01); **m.p.** = 91.6 °C; **$[\alpha]_D^{20}$** = -97.27 ° (c = 0.305 g/100 cm^3, $CHCl_3$); **^{1}H NMR:** (500 MHz, $CDCl_3$, mixture of rotamers) δ [ppm] = 9.30 (bs, 1H, -O*H*), 5.73 (m, 1H, H-10), 5.13 (d, $^3J_{HH}$ = 17.1 Hz, 1H, H-11), 5.07 (d, $^3J_{HH}$ = 10.3 Hz, 1H, H-11'), 4.41 (d, $^3J_{HH}$ = Hz, 0.09H, H-2^{trans}), 4.35 – 4.22 (m, 0.91H, H-2^{cis}), 3.81 – 3.71 (m, 1H, H-5), 3.20 – 3.08 (m, 1H, H-5'), 3.00 – 2.77 (m, 1H, H-4), 2.50 – 2.38 (m, 1H, H-3), 2.08 – 1.82 (m, 1H, H-3'), 1.47/1.42 (2 x s, 9H, H-9); **^{13}C NMR:** (126 MHz, $CDCl_3$, mixture of rotamers) δ [ppm] = 178.7 (C-6), 153.5 (C-7), 136.9 (C-10), 116.5 (C-11), 81.5/80.6 (C-8), 59.1 (C-2), 51.8/51.2 (C-5), 42.1/41.8 (C-4), 36.9/35.0 (C-3), 28.4/28.3 (C-9); **IR:** (ATR) ṽ [cm^{-1}] = 2978 (m), 2946 (w), 2903 (w), 1749 (m), 1699 (s), 1478 (w), 1394 (s), 1367 (s), 1287 (w), 1252 (m), 1159 (s), 1117 (m), 1067 (w), 1057 (w), 997 (w), 916 (m), 863 (w), 767 (w), 735 (w), 655 (w), 593 (w), 547 (w), 500 (w); **MS:** (DIP-EI, 70 eV) m/z (%) = 241 $[M]^+$ (1), 196 $[M-CO_2H]^+$ (3), 140 $[M-Boc]^+$ (74), 96 (65), 68 (26), 57 (100); **HRMS** (ESI): m/z calcd for $C_{12}H_{19}NO_4Na^+$: 264.1206 $[M+Na]^+$; found: 264.1208.

***tert*-butyl-(2*S*,4*R*)-2-((2*S*,5*S*)-2-(tert-butoxycarbonyl)-5-vinylpyrrolidine-1-carbonyl)-4-vinyl-pyrrolidine-1-carboxylate (9)**

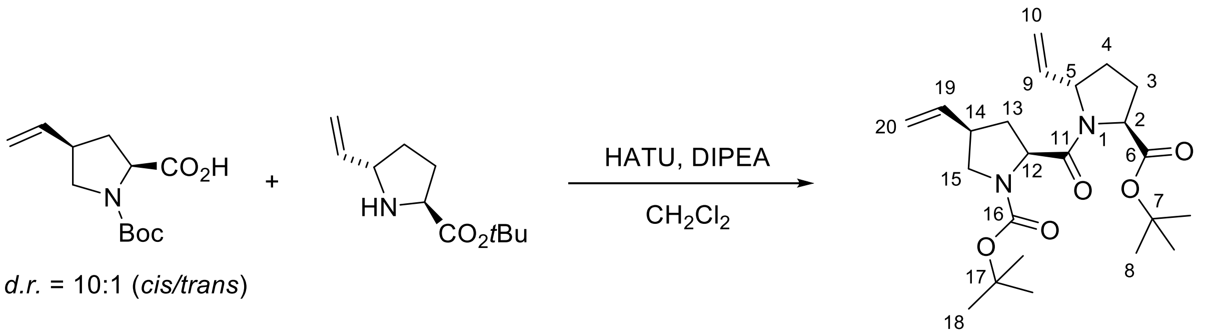

1 **2** **9**

To a solution of 920 mg (3.81 mmol, 1.0 eq.) of a 10:1 (*cis/trans*) mixture of isomers of acid **1** and 0.78 ml (4.6 mmol, 1.2 eq.) DIPEA in 32 ml dry CH_2Cl_2 under inert atmosphere at 0 °C were added 1.89 g (4.96 mmol, 1.3 eq.) HATU, and the mixture was stirred for 1 hour at room temperature. A solution of 903 mg (4.56 mmol, 1.2 eq.) amine **2** in 32 ml dry CH_2Cl_2 was added at 0 °C, and the mixture was stirred for further 16 hours. Once completion of the reaction was determined *via* TLC, 60 ml 1M aqueous HCl were added, and the aqueous layer was extracted twice with each 100 ml CH_2Cl_2. The combined organic layers were dried over $MgSO_4$, the solvent evaporated under reduced pressure and the residue purified *via* column chromatography (SiO_2, EtOAc/cHex 1:7) to afford 1.14 g (2.71 mmol, 71%) of pure product **8** as colorless crystals. **R*f*** = 0.36 (SiO_2, EtOAc/cHex 1:2); **m.p.** = 103.1 °C; **$[\alpha]_D^{20}$** = -53.23 ° (c = 0.340 g/100 cm^3, $CHCl_3$); **^{1}H NMR:** (500 MHz, $CDCl_3$, mixture of rotamers) δ [ppm] = 5.93 – 5.81 (m, 1H, H-9), 5.78 – 5.70 (m, 1H, H-19), 5.40 – 5.15 (m, 2H, H-10), 5.08 (d,

$^3J_{HH}$ = 17.1 Hz, 1H, H-20), 4.99 (dd, $^3J_{HH}$ = 10.4 Hz, $^2J_{HH}$ = 4.7 Hz, 1H, H-20'), 4.52 – 4.37 (m, 3H, H-2, H-5, H-12), 3.78/3.68 (2 x dd, $^2J_{HH}$ = 10.5 Hz, $^3J_{HH}$ = 7.5 Hz, 1H, H-15), 3.23 (t, $^2J_{HH}$ = 10.2 Hz, 1H, H-15'), 2.82 – 2.72 (m, 1H, H-14), 2.49 – 2.40 (m, 1H, H-13), 2.36 – 2.23 (m, 1H, H-4), 2.18 – 2.07 (m, 1H, H-3), 1.91 – 1.74 (m, 3H, H-3', H-4', H-13'), 1.44 – 1.39 (m, 18H, H-8, H-18); **^{13}C NMR:** (126 MHz, $CDCl_3$, mixture of rotamers) δ [ppm] = 171.4/171.3 (C-6, C-11), 154.0/153.9 (C-16), 138.5/138.3 (C-9), 138.2/137.7 (C-19), 116.6 (C-10), 116.0/115.9 (C-20), 81.4/81.1 (C-7), 79.9/79.3 (C-17), 60.5/60.4 (C-2), 59.8/59.5 (C-5), 57.9/57.8 (C-12), 52.3/52.0 (C-15), 42.9/42.0 (C-14), 33.2/35.5 (C-13), 31.6/31.4 (C-4), 28.7/28.2 (C-8, C-18), 26.4/26.3 (C-3); **IR:** (ATR) ṽ [cm^{-1}] = 3082 (w), 2976 (m), 2931 (m), 1750 (m), 1699 (s), 1652 (m), 1478 (w), 1432 (m), 1396 (s), 1367 (m), 1355 (m), 1336 (m), 1293 (w), 1278 (w),1251 (w), 1235 (w), 1217 (w), 1145 (s), 1115 (m), 1077 (w), 1066 (w), 1052 (w), 993 (m), 935 (m), 865 (w), 848 (w), 765 (w), 748 (w), 738 (w), 689 (w), 663 (w), 618 (w), 586 (w), 547 (w), 473 (w); **MS:** (DIP-EI, 70 eV) m/z (%) = 420 [M]$^+$ (1), 392 [M-C_2H_4]$^+$ (2), 364 [M-*t*Bu]$^+$ (3), 347 (5), 319 [M-Boc]$^+$ (4), 308 (9), 291 (13), 263 (11), 196 (9), 140 (100), 96 (82), 57 (69), **HRMS** (ESI): m/z calcd for $C_{23}H_{37}N_2O_5^+$: 421.2697 [M+H]$^+$; found: 421.2698; m/z calcd for $C_{23}H_{36}N_2O_5Na^+$: 443.2516 [M+Na]$^+$; found: 443.2517.

Boc[ProM-5]O*t*Bu

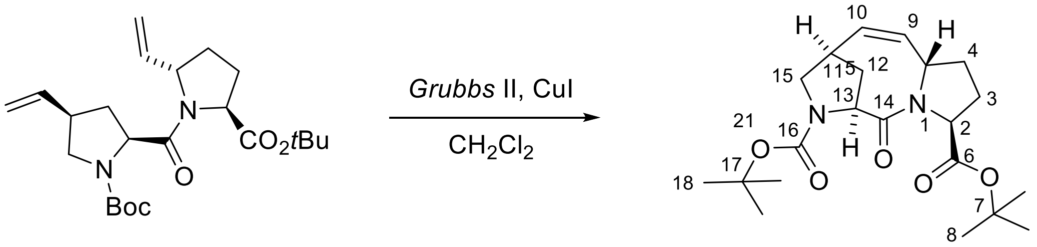

9

Boc**[ProM-5]**O*t*Bu

A solution of 500 mg (1.19 mmol, 1.0 eq.) dipeptide **9**, 68 mg (0.36 mmol, 0.30 eq.) copperiodide and 202 mg (0.238 mmol, 0.2 eq.) *Grubbs* II in dry CH_2Cl_2 under inert atmosphere was heated to 40 °C for 18 hours after which full conversion of the starting material was determined *via* TLC. After cooling to room temperature, three spatulas of each active charcoal and quadrasil were added and the solution filtrated over a celite pad which was washed with EtOAc. The filtrate was concentrated in vacuo and the residue was purified *via* column chromatography (SiO_2, EtOAc/cHex 1:2). The resulting grey solid was dissolved in EtOAc and again 1 spatula of each active charcoal and quadrasil were added. The solution was filtered over celite once more and the solvent evaporated under reduced pressure to yield 407 mg (1.04 mmol, 87%, Lit.[1]: 59%) Boc-protected **ProM 5** *tert*-butyl ester as a colorless solid. **R_f** = 0.19 (SiO_2, EtOAc/cHex 1:1); **m.p.** = 59.6 °C; **$[\alpha]_D^{20}$** = +81.15 ° (c = 0.145 g/100 cm^3, $CHCl_3$); **^{1}H-NMR:** (500 MHz, $CDCl_3$, mixture of rotamers) δ [ppm] = 5.93 – 5.81 (m, 1H, H-10), 5.38 (dd, $^3J_{HH}$ = 11.7 Hz, $^3J_{HH}$ = 7.6 Hz, 1H, H-9), 5.11 – 5.02 (m, 1H, H-5), 4.53 (d, $^3J_{HH}$ = 10.3 Hz, 1H, H-13), 4.32 (d, $^3J_{HH}$ = 9.6 Hz, 1H, H-2), 3.84 (d, $^2J_{HH}$ = 11.3 Hz, 1H, H-15), 3.63 (dd, $^2J_{HH}$ = 11.3 Hz, $^3J_{HH}$ = 7.3 Hz, 1H, H-15'), 2.86 (q, $^3J_{HH}$ = 7.9 Hz, 1H, H-11), 2.45 (ddd, $^2J_{HH}$ = 13.5 Hz, $^3J_{HH}$ = 10.4 Hz, $^3J_{HH}$ = 7.8 Hz, 1H, H-12), 2.23 – 2.14 (m, 1H, H-4), 2.08 – 2.01 (m, 1H, H-3), 1.95 (d, $^2J_{HH}$ = 13.4 Hz, 1H, H-12'), 1.83 – 1.79 (m, 1H, H-3'), 1.72 – 1.68 (m, 1H, H-4'), 1.44 (2 x s, 18H, H-8, H-18); **^{13}C NMR:** (125 MHz, $CDCl_3$, mixture of rotamers) δ [ppm] = 170.9/170.6 (C-6, C-14), 153.7 (C-16), 137.3 (C-10), 134.3 (C-9), 81.1/80.6 (C-7, C-17), 62.7 (C-13), 60.1 (C-2), 53.4 (C-15), 51.9 (C-5), 36.4 (C-12), 35.9 (C-11), 31.9 (C-4), 28.1/27.9 (C-8, C-18), 25.9 (C-3); **IR:** (ATR) ṽ [cm^{-1}] = 2975 (m), 2943 (w), 2886 (w), 1740 (m), 1694 (s), 1638 (s), 1480 (w), 1451 (w), 1388 (s), 1365 (s), 1327 (w), 1304 (w), 1254 (w), 1219 (m), 1148 (s), 1120 (s), 1093 (m), 1050 (w), 999 (w), 912 (w), 894 (w), 834 (w), 813 (w), 790 (w), 772 (m), 736 (w), 655 (w), 606 (w), 569 (w), 569 (w), 519 (w), 543 (w), 470 (w); **MS:** (DIP-EI, 70 eV) m/z (%) = 392 [M]$^+$ (1), 336 [M-*t*Bu]$^+$ (2), 319 (5) 293 (1) [M-Boc]$^+$, 280 (26), 262 (6), 235 (34), 207 (14), 191 (5), 178 (14), 163 (20), 146 (12), 120 (14), 94 (18), 80 (15), 57 (100); **HRMS** (ESI): m/z calcd for $C_{21}H_{33}N_2O_5^+$: 393.2384 [M+H]$^+$; found: 393.2385, m/z calcd for $C_{21}H_{32}N_2O_5Na^+$: 415.2203 [M+Na]$^+$; found: 415.2204.

Fmoc[ProM-5]OH

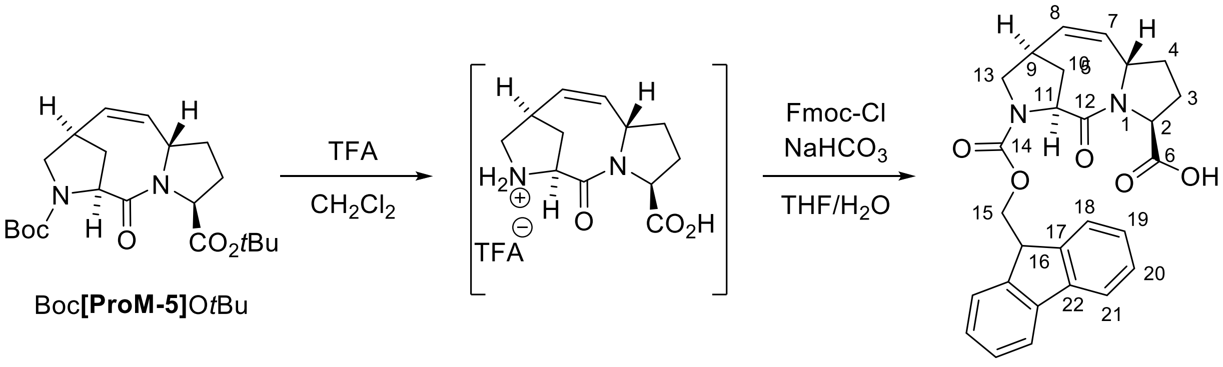

According to a modified procedure by *V. Hack et al.*,[1] 1 ml TFA was added at 0 °C to a solution of 100 mg (0.255 mmol, 1.00 eq.) Boc**[ProM-5]**O*t*Bu in 2 ml dry CH_2Cl_2 at 0 °C. The solution was stirred at room temperature for 1 hour until completion of the deprotection was determined *via* TLC. The solvent was removed under reduced pressure and the residue was taken up in 2 ml sat. aqueous $NaHCO_3$. A solution of 99 mg (0.38 mmol, 1.5 eq.) Fmoc-Cl in 1 ml THF was added dropwise at 0 °C followed by stirring for 16 hours at room temperature. CH_2Cl_2 was added and under stirring at 0 °C the aqueous solution was brought to pH = 1 by addition of 1 M HCl. The aqueous phase was separated and extracted three times with each 20 ml CH_2Cl_2. The combined organic layers were dried over $MgSO_4$, the solvent was evaporated under reduced pressure and the residue purified *via* column chromatography (SiO_2, EtOAc/cHex/MeOH 50:50:1) to afford 104 mg (0.21 mmol, 89%, Lit.[1]: 87%) Fmoc-protected **ProM-5** as a colorless solid. $\mathbf{R_f}$ = 0.09 (SiO_2, EtOAc/cHex/MeOH 5:5:1); **m.p.** = 140.7 °C; $[\alpha]_D^{20}$ = +10.86 ° (c = 0.135 g/100 cm^3, $CHCl_3$); **^{1}H NMR:** (500 MHz, $CDCl_3$, mixture of rotamers) δ [ppm] = 7.76 – 7.69 (m, 3H, H-18, H-21), 7.55 (d, $^3J_{HH}$ = 7.5 Hz, 2H, H-18'), 7.41 – 7.26 (m, 4H, H-19, H-20), 6.08 – 5.54 (m, 1H, H-8), 5.42 – 5.18 (m, 1H, H-7), 5.02/4.78 (2 x t, $^3J_{HH}$ = 7.2 Hz, 1H, H-5), 4.85/4.69 (2 x d, $^3J_{HH}$ = 10.3 Hz, 1H, H-11), 4.59 – 4.46 (m, 2H, H-2, H-15), 4.24 (dd, $^3J_{HH}$ = 9.3 Hz, $^3J_{HH}$ = 6.1 Hz, 1H, H-16), 4.05 – 3.90 (m, 1H, H-15'), 3.89 (d, $^2J_{HH}$ = 11.3 Hz, 1H, H-13), 3.64 (dd, $^2J_{HH}$ = 11.1, $^3J_{HH}$ = 8.0 Hz, 1H, H-13'), 2.83 – 2.75 (m, 1H, H-9), 2.45 – 2.28 (m, 1H, H-10), 2.09 – 1.91 (m, 4H, H-3, H-4, H-10'), 1.67 – 1.57 (m, 1H, H-4'); **^{13}C NMR:** (125 MHz, $CDCl_3$, mixture of rotamers) δ [ppm] = 173.6 (C-6), 172.4 (C-12), 154.3/154.2 (C-14), 145.0/143.4 (C-17), 141.4/141.0 (C-22), 137.4/137.2 (C-8), 133.9/133.8 (C-7), 127.8/127.6 (C-20), 127.4/127.2 (C-19), 126.4/125.4 (C-18), 119.9/119.8 (C-21), 68.8/67.8 (C-15), 63.0/62.7 (C-11), 60.4/60.0 (C-2), 54.0/53.3 (C-13), 52.4/52.4 (C-5), 47.3/46.8 (C-16), 36.9/36.3 (C-10), 35.9/35.3 (C-9), 32.2/31.9 (C-4), 25.5/25.4 (C-3); **IR:** (ATR) ṽ [cm^{-1}] = 2953 (m), 2889 (w), 2602 (w), 1738 (m), 1704 (s), 1637 (m), 1591 (m), 1412 (s), 1347 (m), 1305 (w), 1280 (m), 1170 (m), 1119 (s), 980 (w), 891 (w), 843 (w), 791 (w), 758 (s), 739 (s), 643 (w), 621 (w), 548 (m), 597 (w), 468 (w); **MS:** (DIP-EI, 70 eV) m/z (%) = 223 $[M\text{-}Fmoc]^+$ (1), 178 (100), 165 (12), 152 (27), 139 (2), 126 (2), 89 (6), 76 (9), 63 (5), 50 (2); **HRMS** (ESI): m/z calcd for $C_{27}H_{27}N_2O_5^+$: 459.1914 $[M+H]^+$; found: 459.1918, m/z calcd for $C_{27}H_{26}N_2O_5Na^+$: 481.1734 $[M+Na]^+$; found: 481.1735.

1.3 NMR Spectra of selected compounds

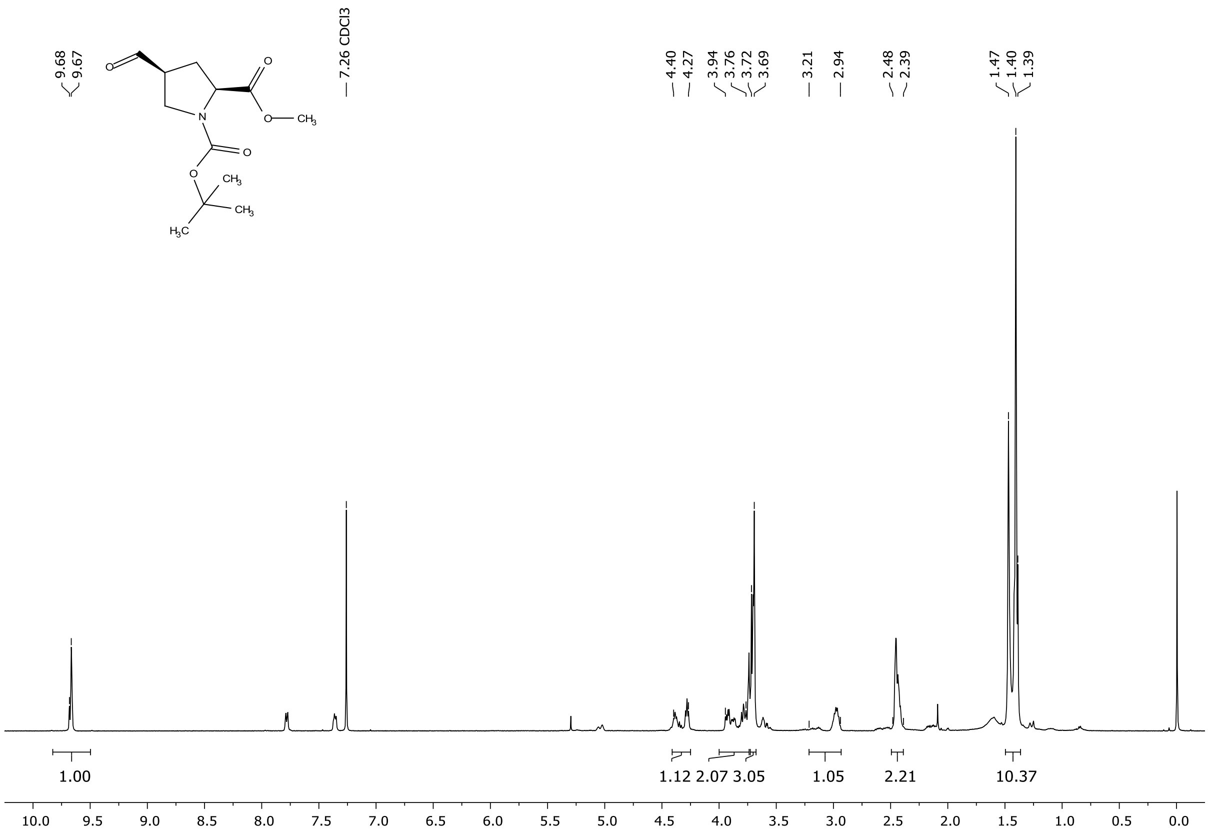

Figure S1: ^{1}H NMR spectrum of 1-(*tert*-butyl)-2-methyl-(2*S*,4*S*)-4-formylpyrrolidine-1,2-dicarboxylate (**7**).

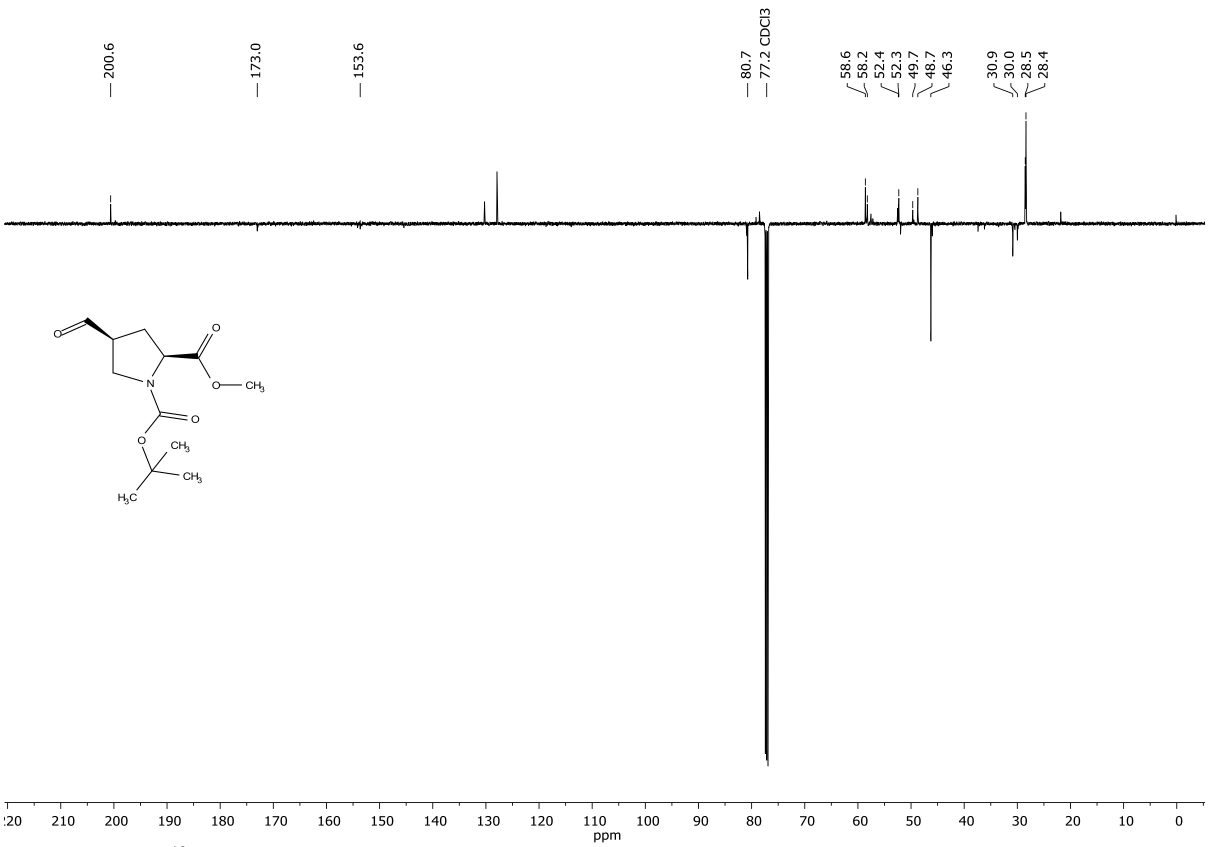

Figure S2: ^{13}C NMR spectrum of 1-(*tert*-butyl)-2-methyl-(2*S*,4*S*)-4-formylpyrrolidine-1,2-dicarboxylate (**7**).

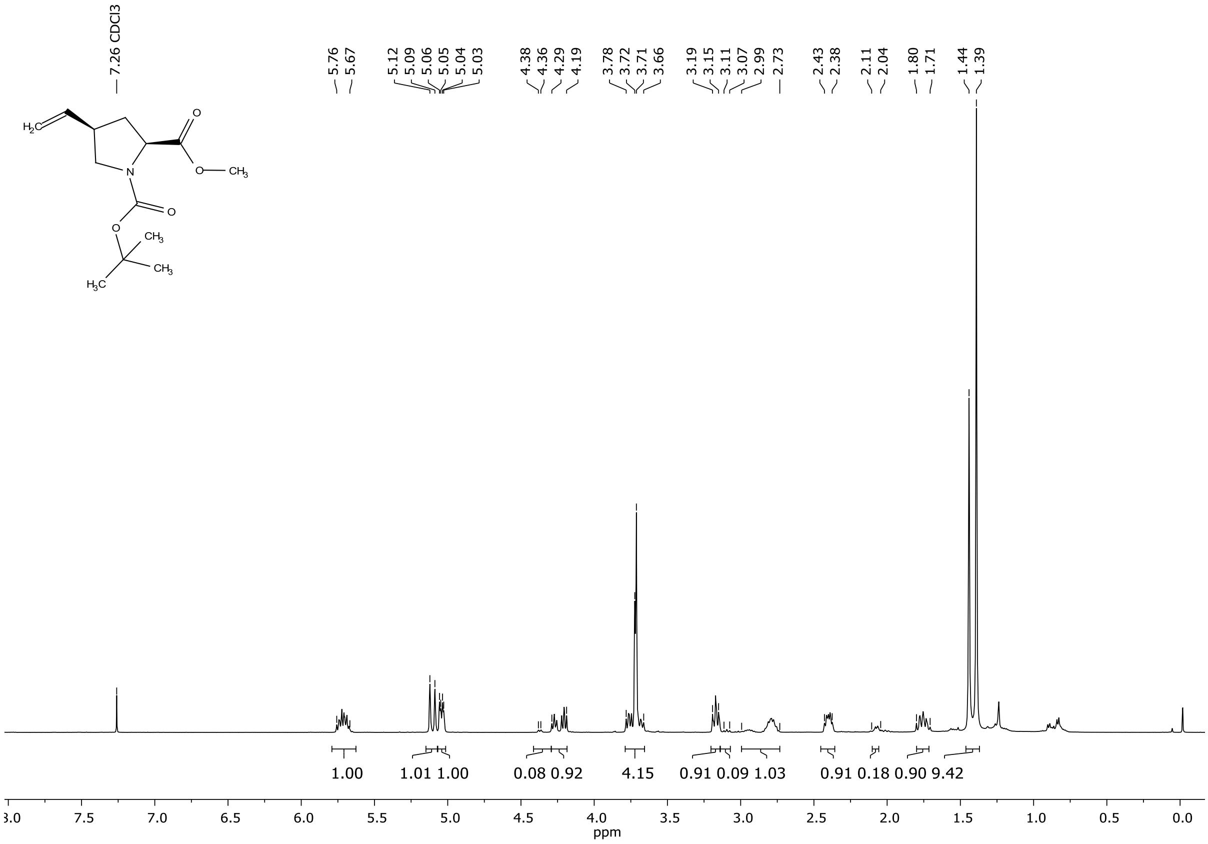

Figure S3: ^{1}H NMR spectrum of 1-(*tert*-butyl)-2-methyl (2*S*,4*R*)-4-vinylpyrrolidine-1,2-dicarboxylate (**8**).

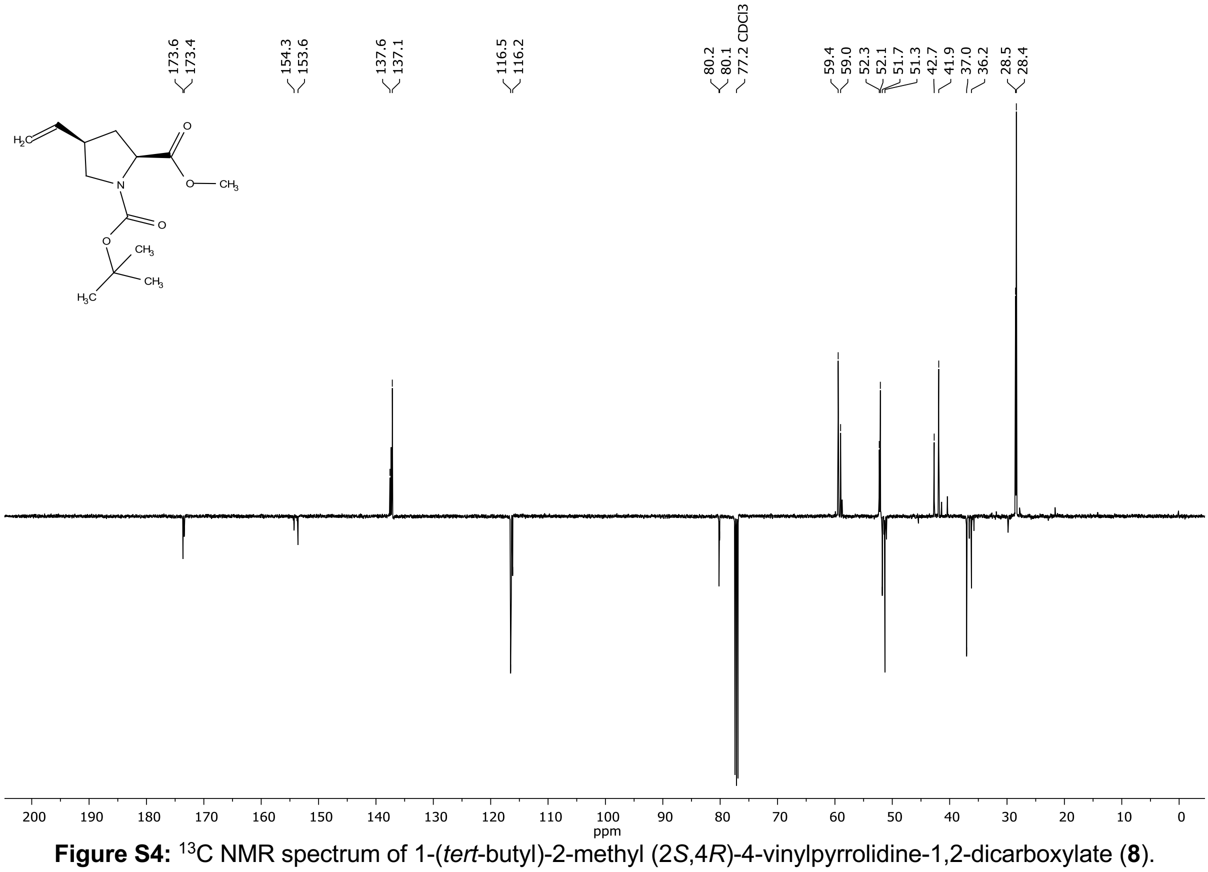

Figure S4: ^{13}C NMR spectrum of 1-(*tert*-butyl)-2-methyl (2*S*,4*R*)-4-vinylpyrrolidine-1,2-dicarboxylate (**8**).

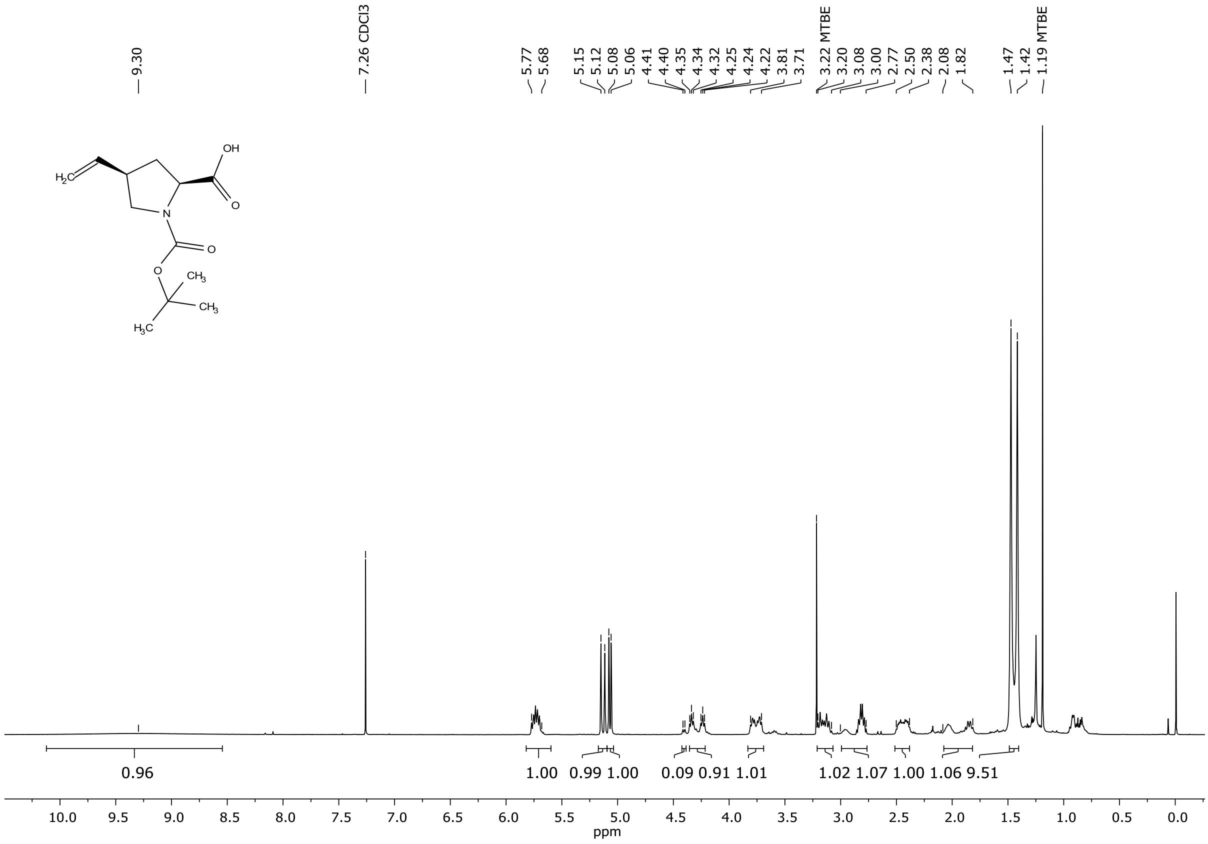

Figure S5: ^{1}H NMR spectrum of (2*S*,4*R*)-1-(*tert*-butoxycarbonyl)-4-vinylpyrrolidine-2-carboxylic acid (**1**).

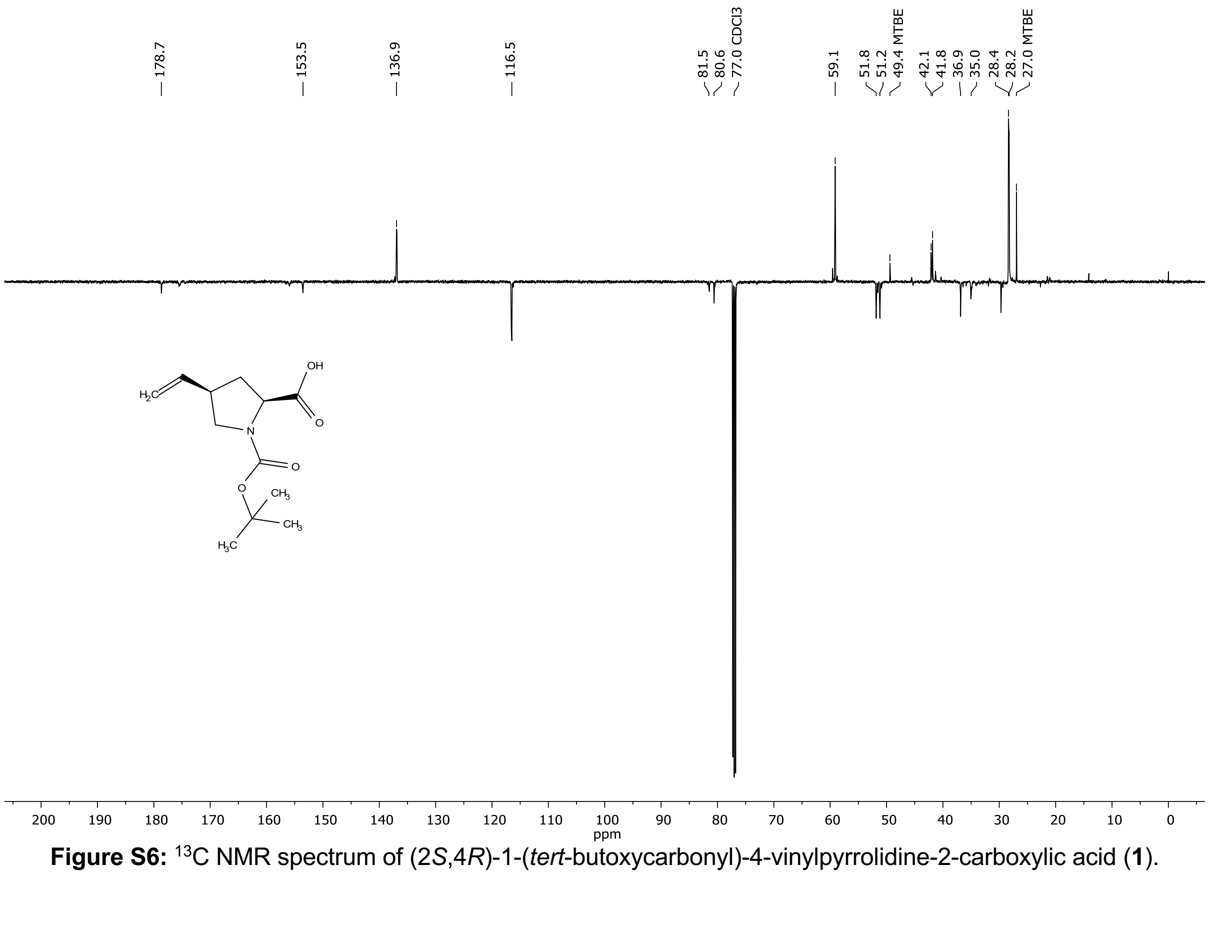

Figure S6: ^{13}C NMR spectrum of (2*S*,4*R*)-1-(*tert*-butoxycarbonyl)-4-vinylpyrrolidine-2-carboxylic acid (**1**).

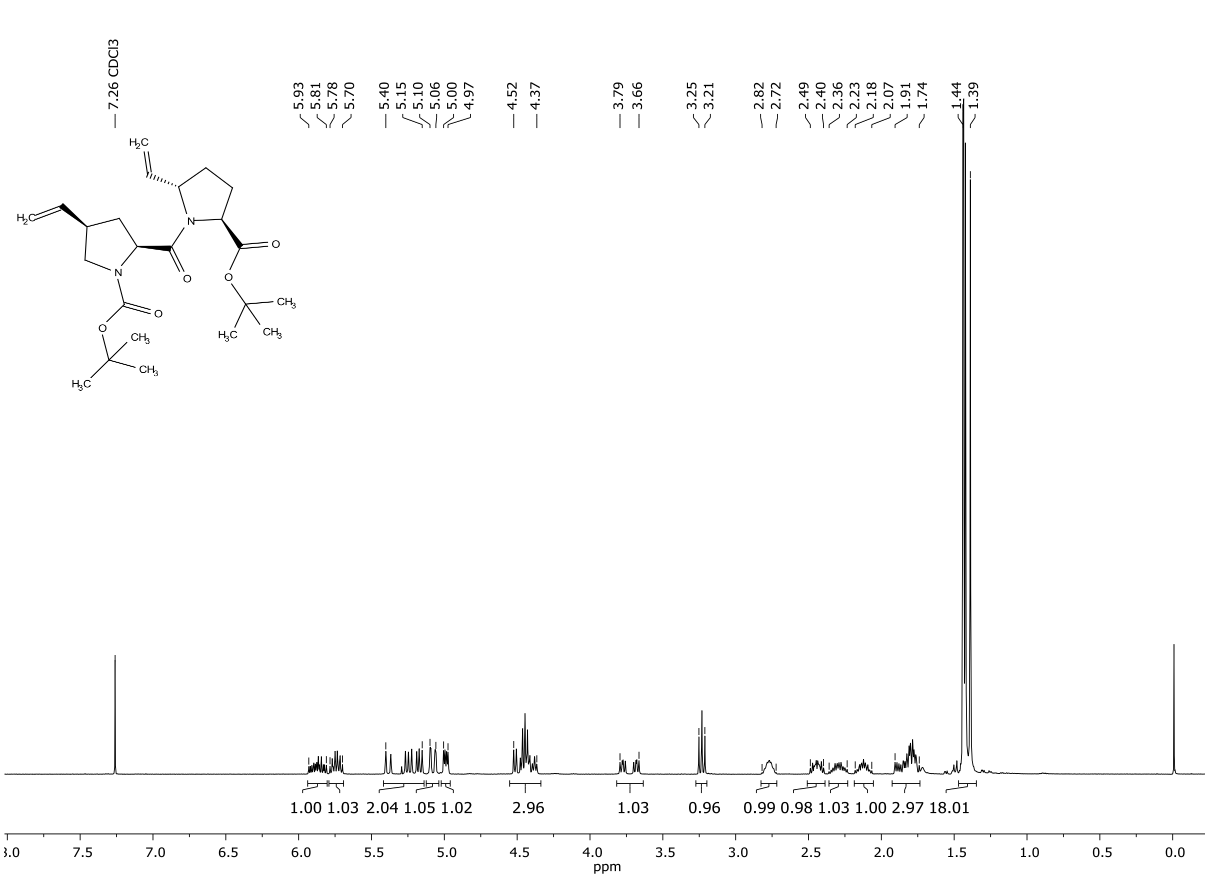

Figure S7: ^{1}H NMR spectrum of *tert*-butyl-(2*S*,4*R*)-2-((2*S*,5*S*)-2-(tert-butoxycarbonyl)-5-vinylpyrrolidine-1-carbonyl)-4-vinylpyrrolidine-1-carboxylate (**9**).

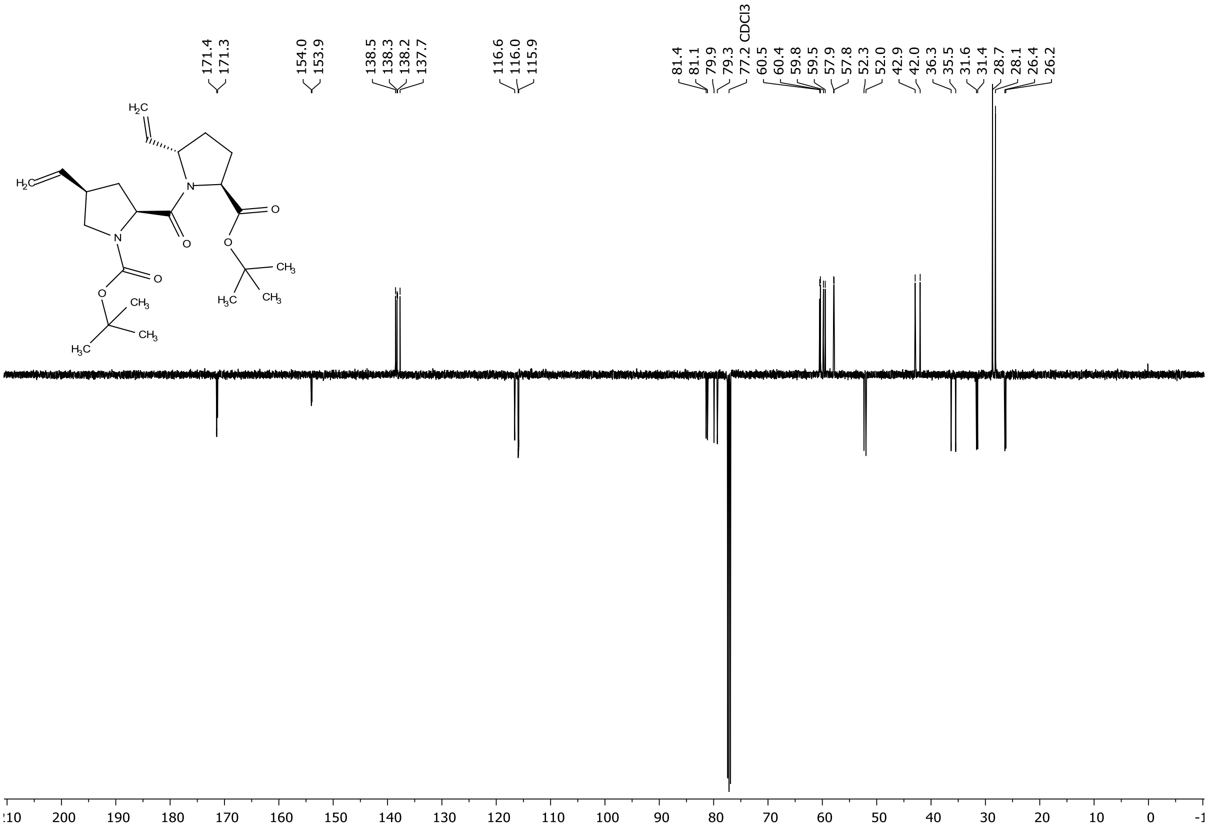

Figure S8: ^{13}C NMR spectrum of *tert*-butyl-(2*S*,4*R*)-2-((2*S*,5*S*)-2-(tert-butoxycarbonyl)-5-vinylpyrrolidine-1-carbonyl)-4-vinylpyrrolidine-1-carboxylate (**9**).

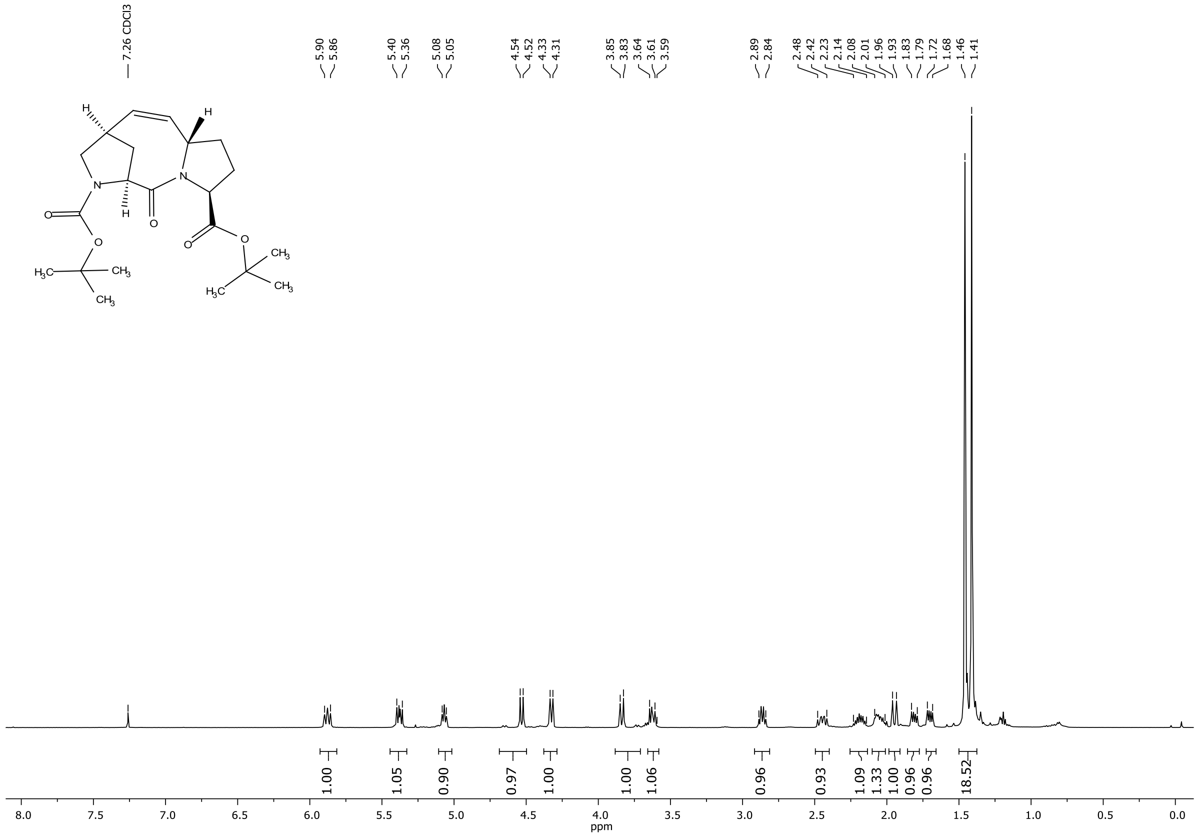

Figure S9: ^{1}H NMR spectrum of **Boc[ProM-5]**O*t*Bu.

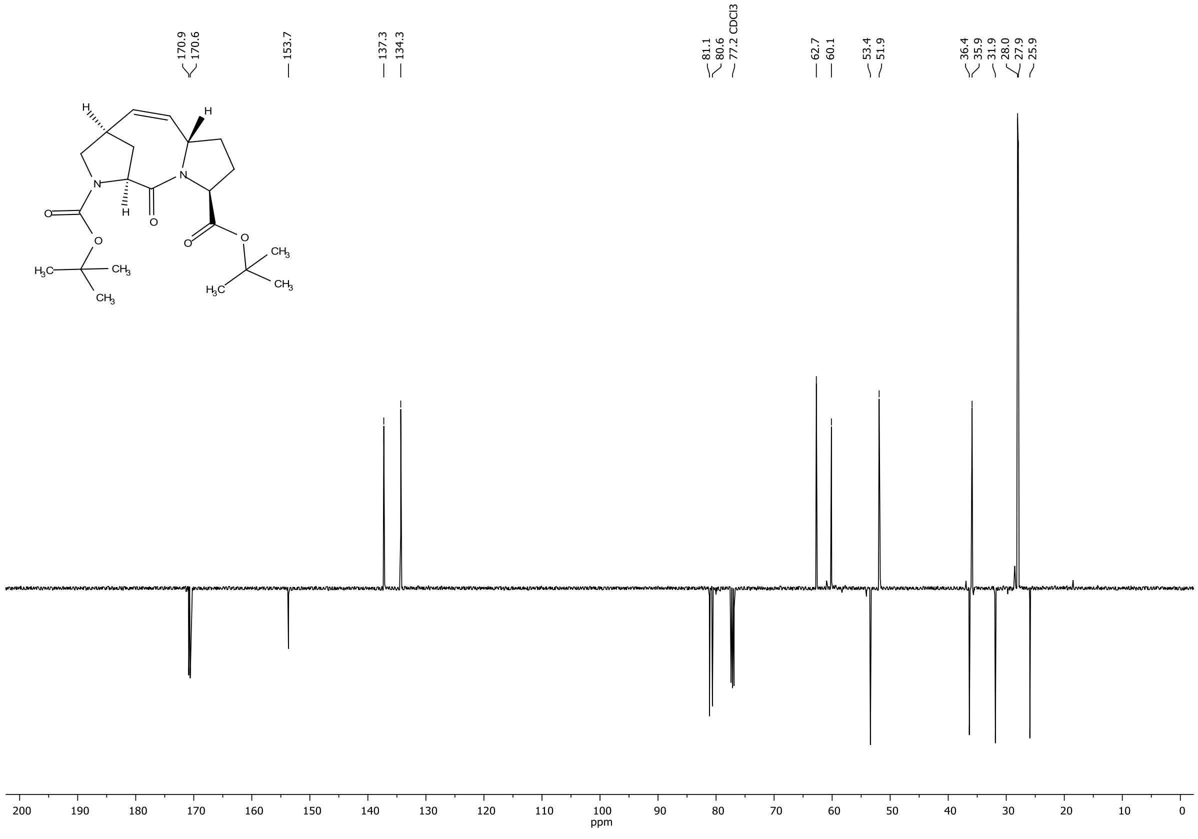

Figure S10: ^{13}C NMR spectrum of Boc**[ProM-5]**O*t*Bu.

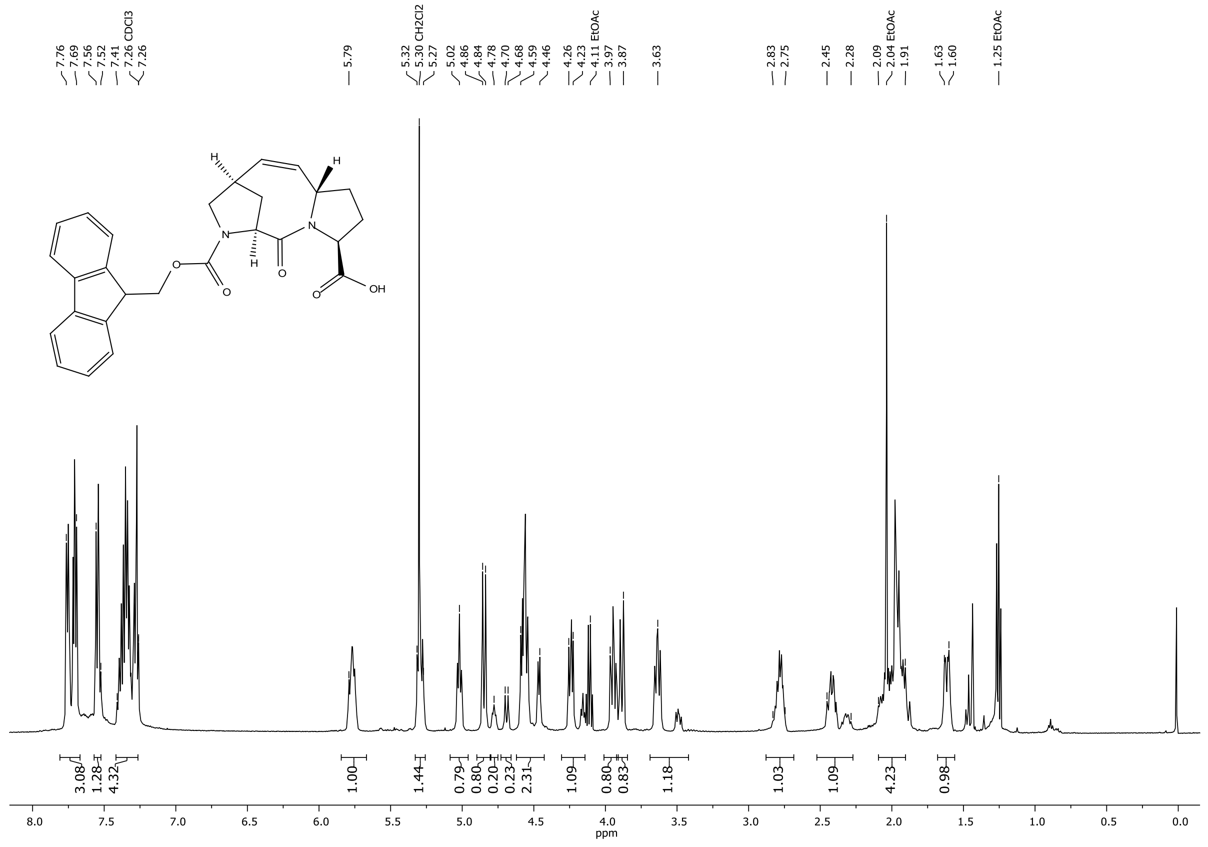

Figure S11: ^{1}H NMR spectrum of Fmoc**[ProM-5]**OH.

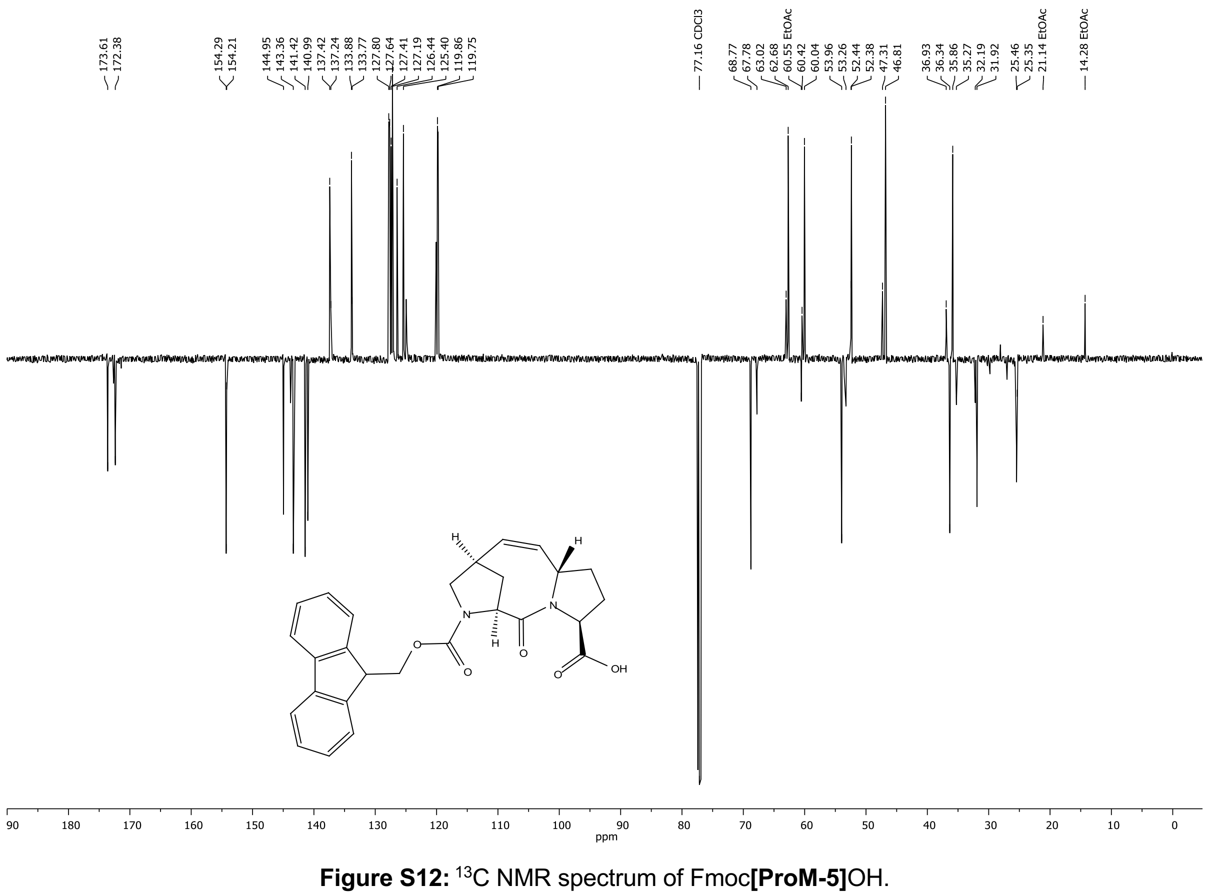

Figure S12: ^{13}C NMR spectrum of Fmoc**[ProM-5]**OH.

1.4 X-Ray crystal structure and parameters of dipeptide 9

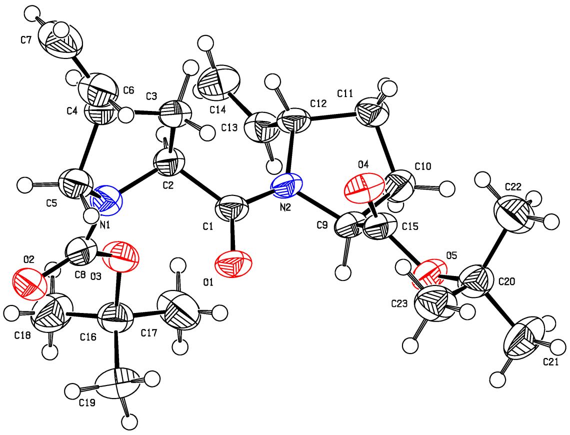

Table S1: Crystal data and structure refinement for dipeptide **9**

Identification code	pme117
Empirical formula	C23 H36 N2 O5
Moiety formula	C23 H36 N2 O5
Formula weight	420.54
Temperature	100(2) K
Wavelength	1.54178 Å
Crystal system	Orthorhombic
Space group	$P2_12_12_1$
Unit cell dimensions	a = 10.8307(4) Å a= 90°.
	b = 11.5240(4) Å b= 90°.
	c = 19.8800(8) Å g = 90°.
Volume	2481.28(16) Å^3
Z	4
Density (calculated)	1.126 Mg/m^3
Absorption coefficient	0.638 mm^{-1}
F(000)	912
Crystal size	0.150 x 0.030 x 0.010 mm^3
Theta range for data collection	4.435 to 72.679°.
Index ranges	-13<=h<=13, -12<=k<=14, -24<=l<=24
Reflections collected	77399
Independent reflections	4924 [R(int) = 0.1451]
Completeness to theta = 67.679°	100.0 %
Absorption correction	Semi-empirical from equivalents
Max. and min. transmission	0.7536 and 0.5676
Refinement method	Full-matrix least-squares on F^2
Data / restraints / parameters	4924 / 0 / 277
Goodness-of-fit on F^2	1.032
Final R indices [I>2sigma(I)]	R1 = 0.0435, wR2 = 0.1051
R indices (all data)	R1 = 0.0558, wR2 = 0.1139
Absolute structure parameter	0.10(12)
Extinction coefficient	n/a
Largest diff. peak and hole	0.193 and -0.197 e.Å^{-3}

2 Peptide synthesis

2.1 Solid Phase Peptide Synthesis (SPPS)

All peptide sequences containing natural amino acids were prepared by a *MultiSynTech* Syro I automated peptide synthesizer *via* solid phase peptides synthesis. For this, 30 mg of rinkamide resin by *Merck* loaded with NovaPEG linker with a surface concentration of 0.48 mmol/g were used, resulting in potential 15 µmol of peptide chains. All automatized coupling reactions were performed in DMF as solvent using equimolar amounts (8.00 eq.) of Fmoc-protected amino acids, diisopropylcarbodiimide (DIC) and ethyl cyanohydroxyiminoacetate (oxyma). Side chain functional groups were protected using acid labile protecting groups. At the end of each coupling cycle a cleavage of the Fmoc protecting group was performed using 30% piperidine in DMF.

Manual coupling of *N*-terminal Fmoc-ProM-5-OH, Fmoc-L-Proline-OH or Fmoc-L-βHAsp-*t*Bu-OH to peptide sequences synthesized as described above was performed by adding a solution of Fmoc-protected amino acid (2.0 eq.), [1-[bis(dimethylamino)methylene]-1H-1,2,3-triazolo-[4,5-b]-pyridinium 3-oxide hexafluorophosphate (HATU) (2.00 eq.) and *NN*-diisopropylethylamine (DIPEA) (2.00 eq.) in 300 µl DMF/CH_2Cl_2 (9:1) to the resin containing the respective peptide. The resin was shaken at room temperature for 2 hours, washed with DMF, CH_2Cl_2, MeOH and Et_2O and dried. To determine coupling success, a *Kaiser* test was performed. One drop each of a solution of ninhydrin in ethanol (0.05 g/ml), a solution of phenol in ethanol solution (4 g/mL) and a solution of potassium cyanide in pyridine (0.02 mM) was added to the dried resin. The resin was then incubated at 95 °C for 5 min upon which a color change to blue indicates the presence of free amino functions and therefore an incomplete coupling. Upon complete coupling an Fmoc deprotecting was performed *via* stirring the resins in 30% piperidine in DMF, before the next coupling could be performed.

After a completed peptide synthesis, the free *N*-terminus was acetylatedby addition of 20 eq. acetic anhydride and 20 eq. DIPEA in 300 µl CH_2Cl_2 to the resin, followed by shaking for 30 min at room temperature and then washing with DMF, CH_2Cl_2, MeOH and Et_2O.

The peptides were cleaved from the resin by addition of 1 ml a mixture of trifluoro acetic acid (TFA), triisopropylsilane and water (95:2.5:2.5) and shaking at room temperature for 3 hours. The resin was filtered from the solution and washed with 0.2 mL TFA and the combined filtrates were added to 10 ml cold Et_2O and stored at -20 °C for 16 hours, upon which the peptides precipitated. They were then washed several times with cold Et_2O through centrifugation, dissolved in *t*BuOH/water (1:4) and lyophilized. For purification, preparative RP-HPLC using a *Hitachi Elite* LaChrom system with a *Macherey Nagel* VP 250/8 Nucleodur 100-5 C18ec column was used. As solvents 0.1% aqueous TFA and acetonitrile were used with a linear gradient (30%→60% acetonitrile over 30 minutes) and a flow rate of 1.5 ml/min. The acetonitrile was removed from all relevant fractions using a *Horizon Technology* Xcel Vap in an air flow gradient from 880 to 1640 mbar in 20 minutes at 65 °C before lyophilization. The product identity was confirmed by LC-ESI-MS analysis using a *Merck* Chromolith Performance RP-18e endcapped 100-4.6 mm HPLC column coupled to a *ThermoScientific* LTQ-XL linear ion trap mass spectrometer (gradient: 20%→70% acetonitrile in 0.1% aqueous formic acid over 15 minutes). Purities were determined *via* integration of peaks in the UV chromatogram.

2.2 Characterization by Circular Dichroism (CD)

Circular dichroism spectra were recorded using a J715 Spectropolarimeter by *JASCO* at wavelengths from 180 to 260 nm in steps of 0.2 nm. For each spectrum 4 sets of measurements were recorded, and a baseline subtraction of the solvent was performed. A quartz cuvette with a thickness of 1 mm was used. Before measurement solutions of peptides in the respective measurement medium were prepared in concentrations of 60 µM. As medium 10 mM phosphate buffer at pH = 7.4 was used. The measured ellipticity was converted into the mean residue ellipticity for number of residues per molecule of 22 using the formula:

$$[\theta] = \frac{\theta}{(10*n*c*d)} \ [\frac{deg*cm^2}{dmol}]$$

Obtained spectra were smoothed using the web server-based plotting tool CAPITO[2] applying a Savitzky-Golay filter and plotted using the software *Origin* by OriginLabs. The raw data was analyzed by the online-tool DichroWeb[3] using the CDSSTR method to determine fractional α-helicities.

2.3 Summary and LC-MS Spectra of synthesized peptides

#	Peptide sequence	M []	Purity	Yield
P-1-1	Ac-**βHAsp-[ProM-5]**-QAKTFLDKFNHEAEDLFYQ- NH_2	2733.9	93%	18%
P-1-2	Ac-**βHAsp**-**P**-**P**-QAKTFLDKFNHEAEDLFYQ- NH_2	2709.9	98%	21%
P-1-3	Ac-QAKTFLDKFNHEAEDLFYQ-NH_2	2386.9	98%	71%
P-2-1	Ac-**βHAsp-[ProM-5]**-EQAKTFLDKFNHEAEDLFYQK- NH_2	2991.4	92%	18%
P-2-2	Ac-**βHAsp**-**P**-**P**-EQAKTFLDKFNHEAEDLFYQK- NH_2	2967.6	96%	62%
P-2-3	Ac-EQAKTFLDKFNHEAEDLFYQK-NH_2	2644.2	91%	26%
P-3-1	Ac-**βHAsp-[ProM-5]**-EQAKAAADKAAHEAEAAAYQK-NH_2	2562.3	83%	30%
P-3-2	Ac-**βHAsp**-**P**-**P**-EQAKAAADKAAHEAEAAAYQK-NH_2	2538.1	88%	45%
P-3-3	Ac-EQAKAAADKAAHEAEAAAYQK-NH_2	2214.7	99%	8%
P-4	Ac-**βHAsp**-**P**-**P**-AQHAAEAAAEQEYAKADAKKA-NH_2	2538.1	95%	11%
P-5	Ac-**βHAsp**-**P**-**P**-EQAKAAADKAAHE-NH_2	1705.1	92%	12%
P-6	Ac-**βHAsp**-**P**-**P**-EQAKAAADKAAHEAEAAAYQAAL-NH_2	2665.2	81%	28%

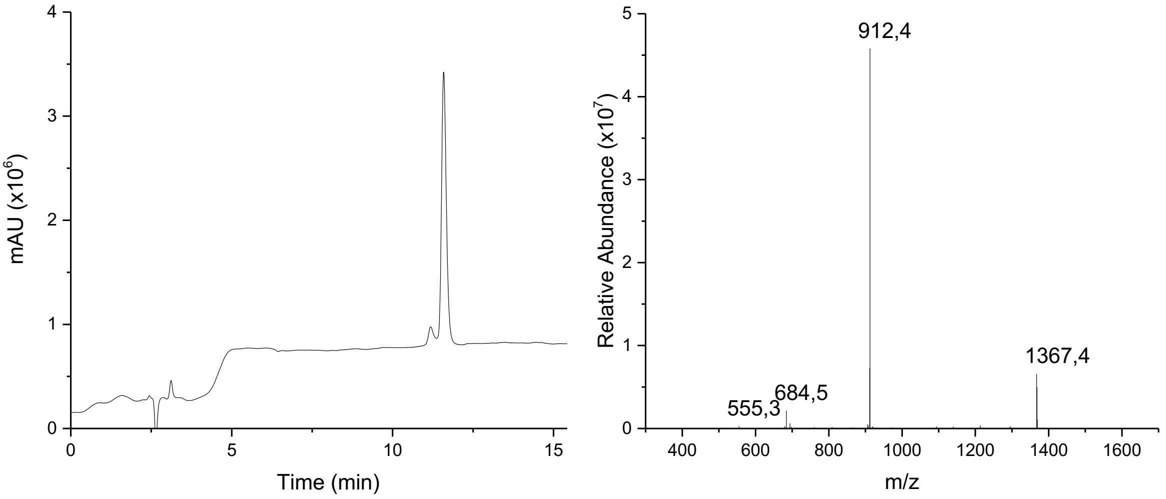

MS (ESI):	$[M+2H]^{2+}$	$[M+3H]^{3+}$	$[M+4H]^{4+}$	$[M+3H+2NH_3]^{5+}$
m/z calc. for $C_{127}H_{177}N_{29}O_{39}$	1367.4	912.3	684.5	554.6
found	1367.8	912.4	684.5	555.3

Figure S13: LC-ESI-MS analysis of P-1-1. Left: UV chromatogram. Right: Mass spectrum at 11.7 minutes. Bottom: Comparison of measured m/z to calculated values.

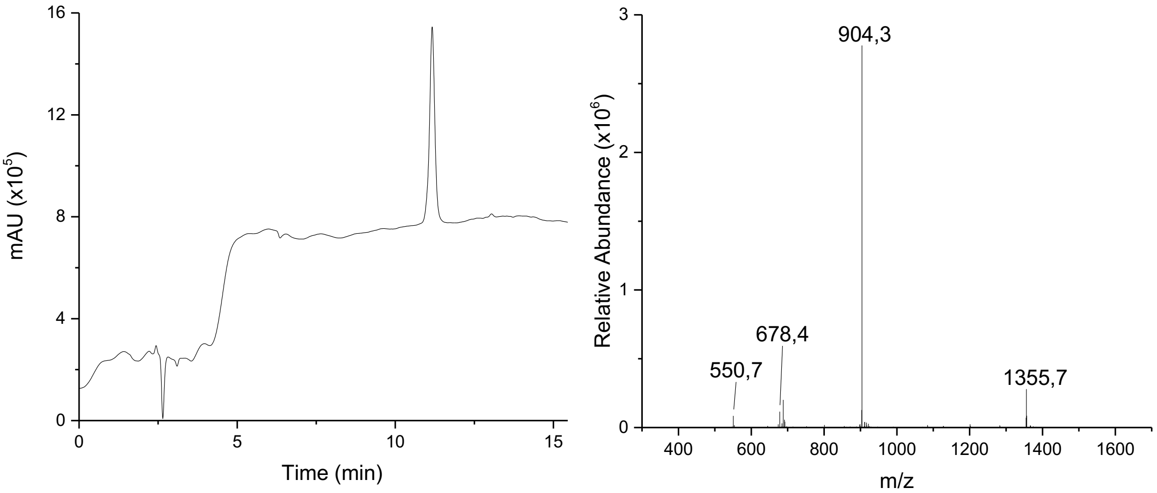

MS (ESI):	$[M+2H]^{2+}$	$[M+3H]^{3+}$	$[M+4H]^{4+}$	$[M+3H+2NH_3]^{5+}$
m/z calc. for $C_{125}H_{177}N_{29}O_{39}$	1356.0	904.3	678.5	549.8
found	1355.7	904.3	678.4	550.7

Figure S14: LC-ESI-MS analysis of P-1-2. Left: UV chromatogram. Right: Mass spectrum at 11.3 minutes. Bottom: Comparison of measured m/z to calculated values.

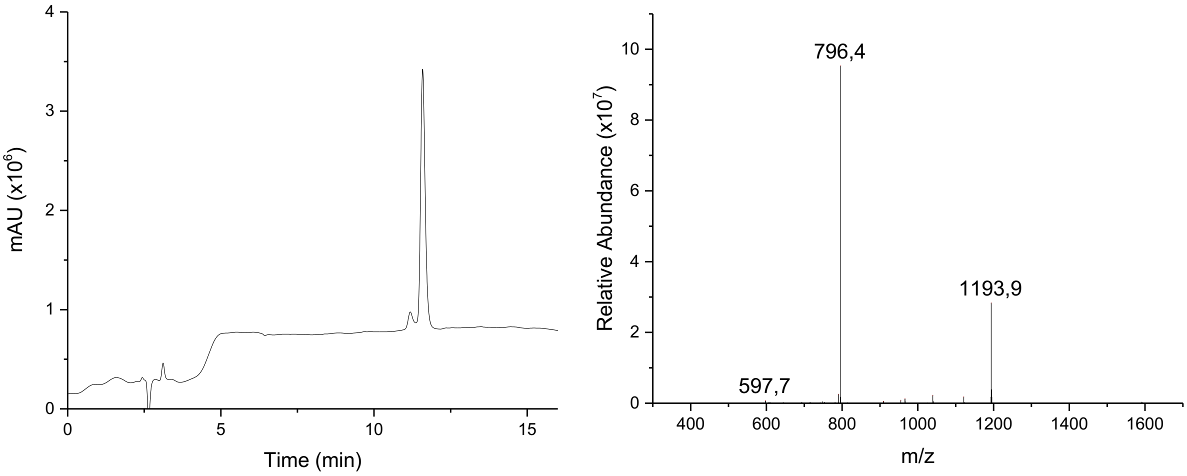

MS (ESI):	$[M+2H]^{2+}$	$[M+3H]^{3+}$	$[M+4H]^{4+}$
m/z calc. for $C_{127}H_{177}N_{29}O_{39}$	1194.5	796.6	597.7
found	1193.9	796.4	597.7

Figure S15: LC-ESI-MS analysis of P-1-3. Left: UV chromatogram. Right: Mass spectrum at 10.9 minutes. Bottom: Comparison of measured m/z to calculated values.

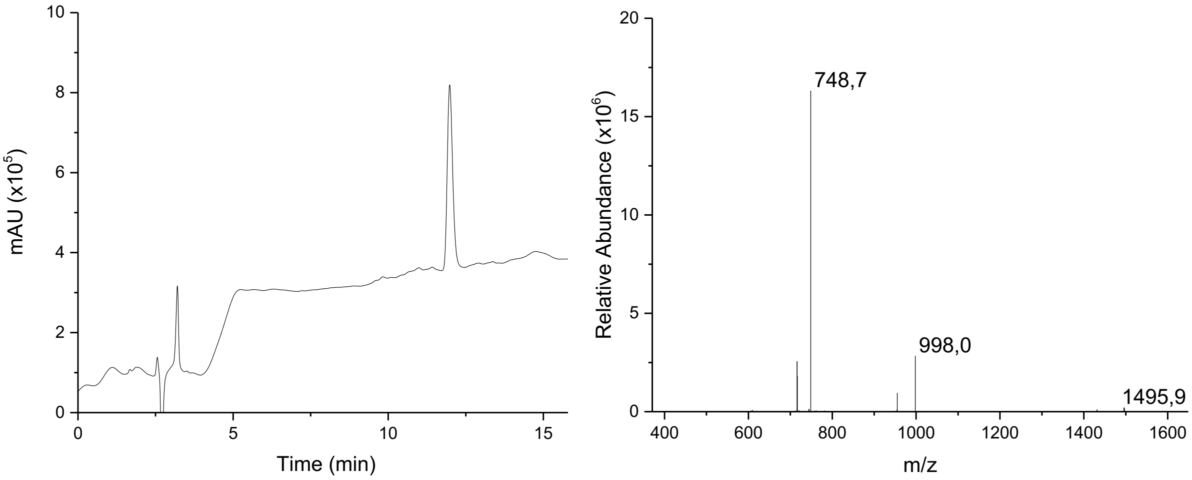

MS (ESI):	$[M+2H]^{2+}$	$[M+3H]^{3+}$	$[M+4H]^{4+}$
m/z calc. for $C_{127}H_{177}N_{29}O_{39}$	1496.7	998.1	748.8
found	1495.9	998.0	748.8

Figure S16: LC-ESI-MS analysis of P-2-1. Left: UV chromatogram. Right: Mass spectrum at 12.0 minutes. Bottom: Comparison of measured m/z to calculated values.

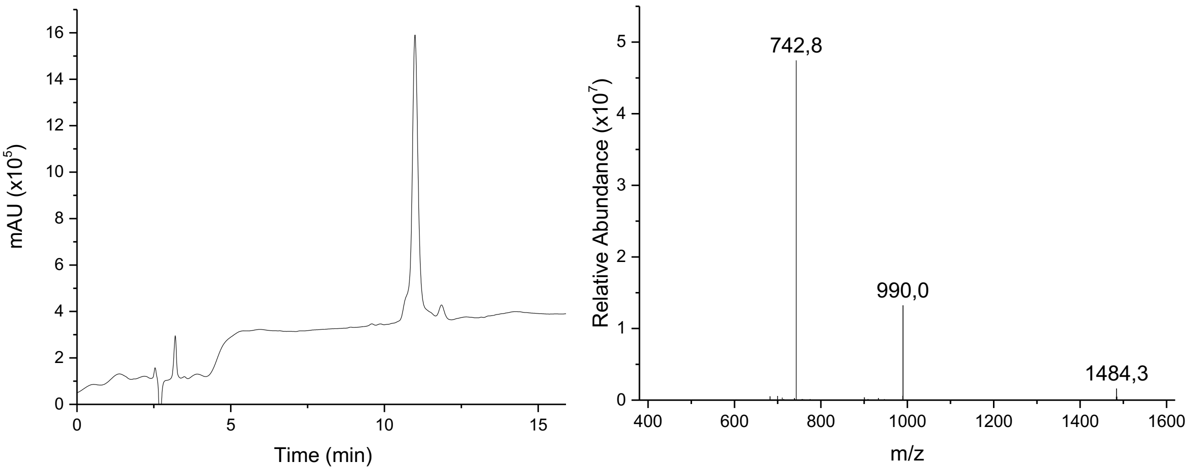

MS (ESI):	$[M+2H]^{2+}$	$[M+3H]^{3+}$	$[M+4H]^{4+}$
m/z calc. for $C_{127}H_{177}N_{29}O_{39}$	1484.8	990.2	742.9
found	1484.4	990.0	742.8

Figure S17: LC-ESI-MS analysis of P-2-2. Left: UV chromatogram. Right: Mass spectrum at 11.0 minutes. Bottom: Comparison of measured m/z to calculated values.

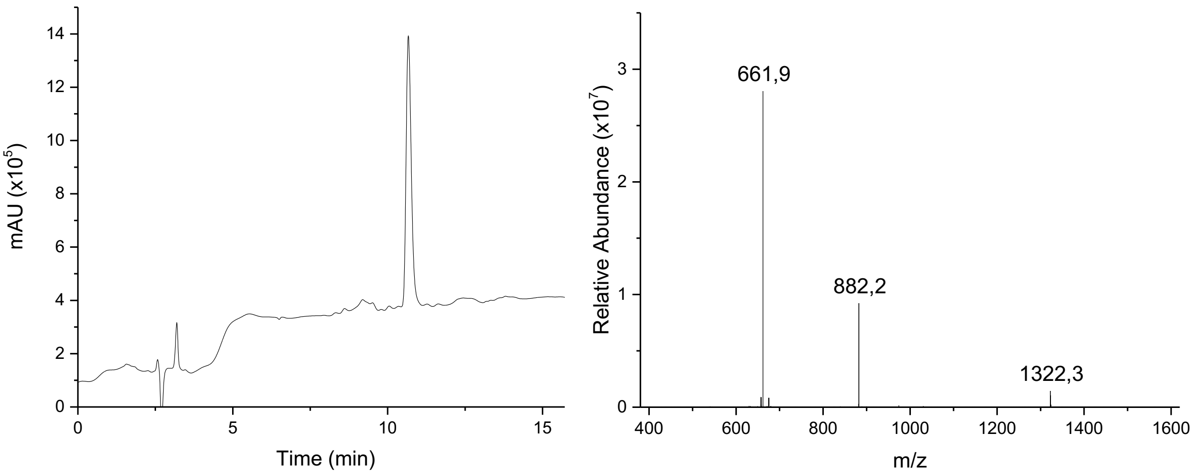

MS (ESI):	$[M+2H]^{2+}$	$[M+3H]^{3+}$	$[M+4H]^{4+}$
m/z calc. for $C_{127}H_{177}N_{29}O_{39}$	1323.1	882.4	662.1
found	1322.2	882.2	661.9

Figure S18: LC-ESI-MS analysis of P-2-3. Left: UV chromatogram. Right: Mass spectrum at 10.7 minutes. Bottom: Comparison of measured m/z to calculated values.

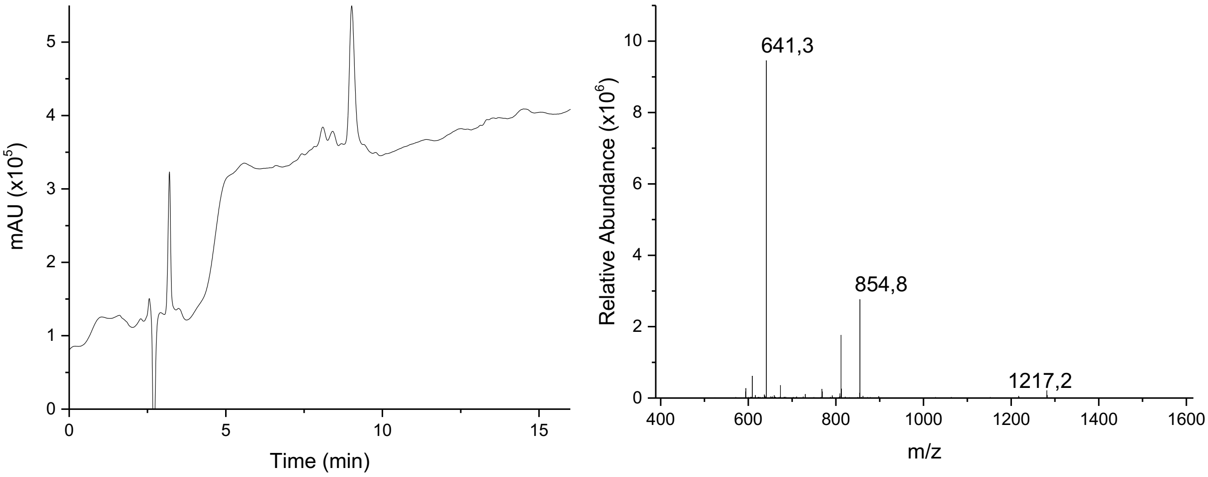

MS (ESI):	$[M+2H]^{2+}$	$[M+3H]^{3+}$	$[M+4H]^{4+}$
m/z calc. for $C_{127}H_{177}N_{29}O_{39}$	1282.2	855.1	641.6
found	1281.2	854.8	641.3

Figure S19: LC-ESI-MS analysis of P-3-1. Left: UV chromatogram. Right: Mass spectrum at 9.0 minutes. Bottom: Comparison of measured m/z to calculated values.

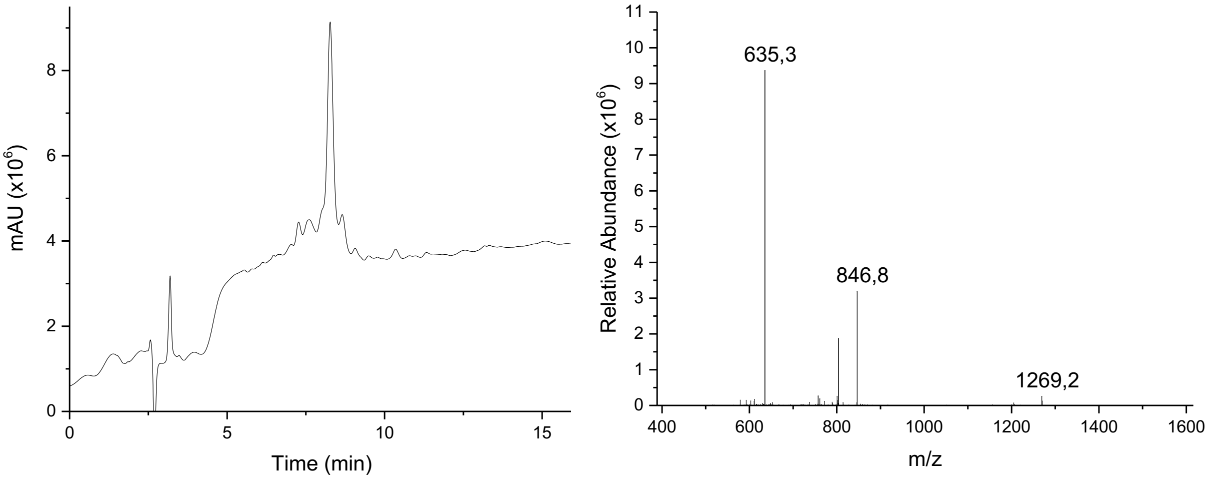

MS (ESI):	$[M+2H]^{2+}$	$[M+3H]^{3+}$	$[M+4H]^{4+}$
m/z calc. for $C_{127}H_{177}N_{29}O_{39}$	1270.0	847.0	635.5
found	1269.2	846.8	635.3

Figure S20: LC-ESI-MS analysis of P-3-2. Left: UV chromatogram. Right: Mass spectrum at 8.3 minutes. Bottom: Comparison of measured m/z to calculated values.

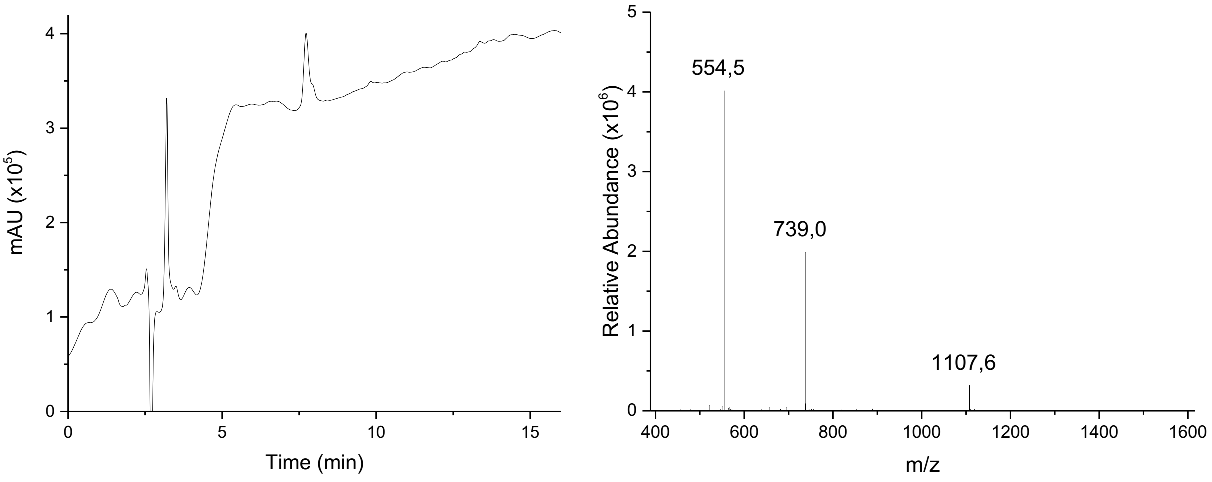

MS (ESI):	$[M+2H]^{2+}$	$[M+3H]^{3+}$	$[M+4H]^{4+}$
m/z calc. for $C_{127}H_{177}N_{29}O_{39}$	1108.3	739.2	554.7
found	1107.6	739.0	554.5

Figure S21: LC-ESI-MS analysis of P-3-3. Left: UV chromatogram. Right: Mass spectrum at 7.7 minutes. Bottom: Comparison of measured m/z to calculated values.

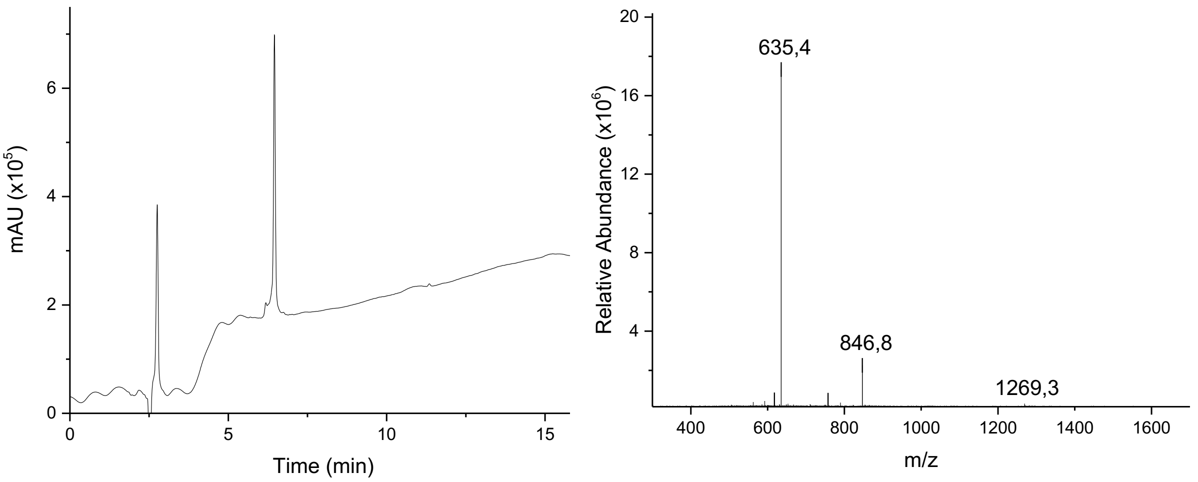

MS (ESI):	$[M+2H]^{2+}$	$[M+3H]^{3+}$	$[M+4H]^{4+}$
m/z calc. for $C_{127}H_{177}N_{29}O_{39}$	1270.0	847.0	635.5
found	1269.3	846.8	635.4

Figure S22: LC-ESI-MS analysis of P-4. Left: UV chromatogram. Right: Mass spectrum at 6.5 minutes. Bottom: Comparison of measured m/z to calculated values.

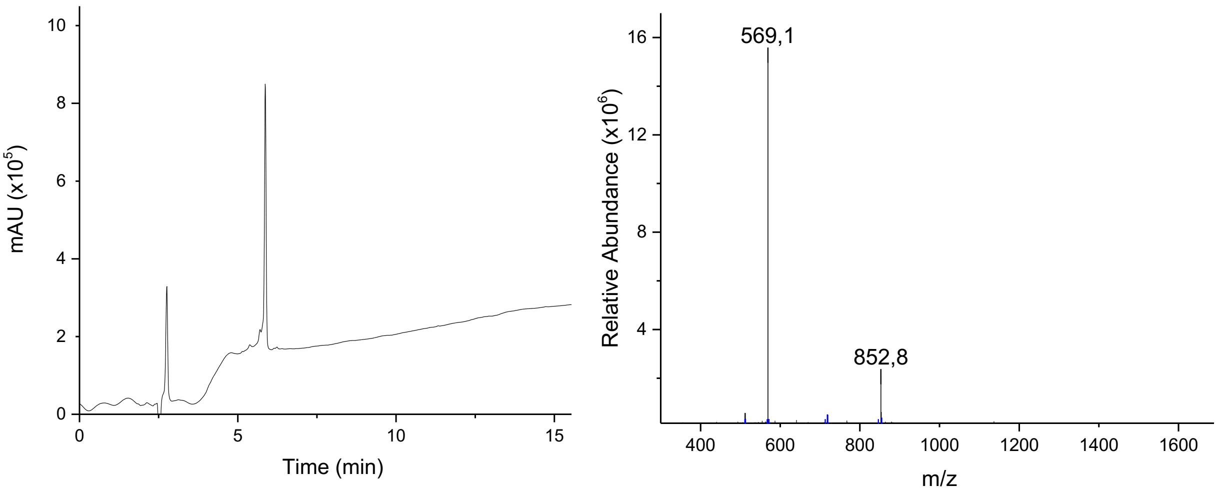

MS (ESI):	$[M+2H]^{2+}$	$[M+3H]^{3+}$
m/z calc. for $C_{127}H_{177}N_{29}O_{39}$	853.5	569.4
found	852.8	569.1

Figure S23: LC-ESI-MS analysis of P-5. Left: UV chromatogram. Right: Mass spectrum at 5.9 minutes. Bottom: Comparison of measured m/z to calculated values.

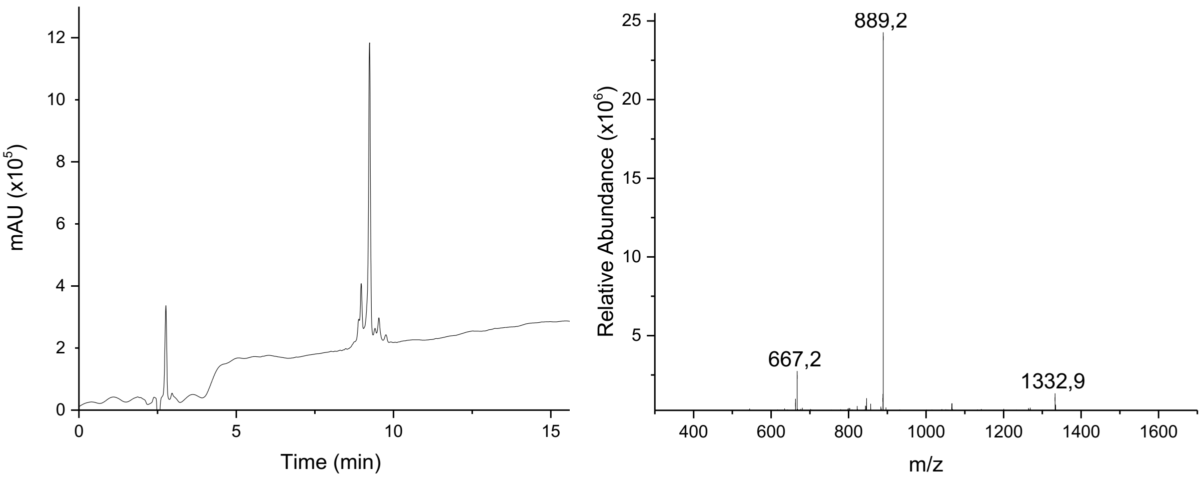

MS (ESI):	$[M+2H]^{2+}$	$[M+3H]^{3+}$	$[M+4H]^{4+}$
m/z calc. for $C_{127}H_{177}N_{29}O_{39}$	1333.6	889.4	667.3
found	1332.9	889.2	667.2

Figure S24: LC-ESI-MS analysis of P-6. Left: UV chromatogram. Right: Mass spectrum at 9.3 minutes. Bottom: Comparison of measured m/z to calculated values.

3 Predictions of α-helicities

The design of new amino acid sequences for the synthesis of optimized peptides with increased helicity, the online tool AGADIR[4] was used. As conditions for the calculations a temperature of 277 K, a pH of 7 and an ionic strength of 0.1 were chosen. As starting sequence peptide P-1-2 was chosen and non-binding amino acids were stepwise replaced by alanine, to improve helical folding properties. Since only canonical amino acids can be chosen in AGADIR, β-Homoaspartic acid was replaced by glutamic acid for the calculations. All peptides were calculated wit acetylated *N*-terminus and amidated *C*-terminus.

Table S1: AGADIR calculations for the optimization of the peptide sequence. Highlighted residues were believed to be necessary for binding and not altered, other amino acids were replaced stepwise by alanine to increase helical content from P-2-2 (entry 1) to P-3-2 (entry 9). Further, predictions of *C*- and *N*-terminal modifications to the original sequence P-1-2 (entry 1) as well as the modified sequence P-3-1 (entry 8) were performed.

#	Sequence	Predicted Helicity*
1	Ac-**EPP**E**Q**AKTFL**DK**FN**H**EA**E**DLA**YQ**K-NH_2	1.59%
2	Ac-**EPP**E**Q**AKTFL**DK**FN**H**EA**E**DAA**YQ**K-NH_2	1.72%
3	Ac-**EPP**E**Q**AKTFL**DK**FN**H**EA**E**AAA**YQ**K-NH_2	3.48%
4	Ac-EPPEQAKTFLDKFNHAAEAAAYQK-NH_2	3.22%
5	Ac-**EPP**E**Q**AKTFL**DK**FA**H**EA**E**AAA**YQ**K-NH_2	6.13%
6	Ac-**EPP**E**Q**AKTFL**DK**AA**H**EA**E**AAA**YQ**K-NH_2	9.57%
7	Ac-**EPP**E**Q**AKTFA**DK**AA**H**EA**E**AAA**YQ**K-NH_2	11.86%
8	Ac-**EPP**E**Q**AKTAA**DK**AA**H**EA**E**AAA**YQ**K-NH_2	17.33%
9	Ac-**EPP**E**Q**AKAAA**DK**AA**H**EA**E**AAA**YQ**K-NH_2	26.23%
10	Ac-**EPP**E**Q**AAAAA**DK**AA**H**EA**E**AAA**YQ**K-NH_2	25.30%
11	Ac-**EPPQ**AKTFL**DK**FN**H**EAEDLF**YQ**-NH_2	0.78%
12	Ac-**EPPQ**AKTFL**DK**FN**H**EAEDLF**YQ**K-NH_2	0.89%
13	Ac-**EPP**E**Q**AKTFL**DK**FN**H**EAEDLF**YQ**-NH_2	0.98%
14	Ac-**EPP**E**Q**AKTFL**DK**FN**H**EAEDLF**YQ**K-NH_2	1.05%
15	Ac-**EPPQ**AKAAA**DK**AA**H**EAEAAA**YQ**-NH_2	16.08%
16	Ac-**EPPQ**AKAAA**DK**AA**H**EAEAAA**YQ**K-NH_2	19.36%
17	Ac-**EPP**E**Q**AKAAA**DK**AA**H**EAEAAA**YQ**-NH_2	22.43%
18	Ac-**EPP**E**Q**AKAAA**DK**AA**H**EAEAAA**YQ**K-NH_2	26.23%

*Helix content values predicted by the algorithm AGADIR at 277 K and pH = 7.

4 Helical Wheel Plots

Helical Wheels were drawn for helices resulting from βHAsp-PP coupled peptides (P-1-2, P-2-2, P-3-2) and reference peptides containing the corresponding parent amino acid sequences (P-1-3, P-2-3, P-3-3) to investigate the effect of the N-cap. The plots were produced using the online tool EMBOSS: pepwheel[5] with 18 steps and 5 turns. Polar or negatively charged residues are shown as red diamonds, positively charged residues as black octagons, residues with aliphatic side chains as blue squares and other hydrophobic residues in purple. β-Homoaspartic acid was replaced by glutamic acid for these plots.

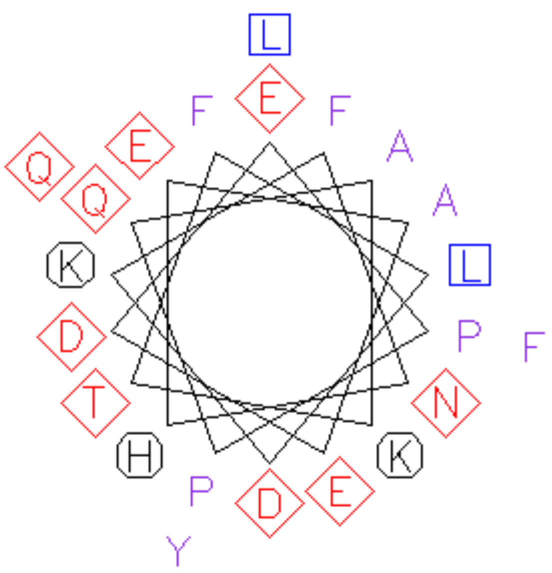

Figure S25: Left: Helical Wheel of P-1-2; Right: Helical Wheel of P-1-3.

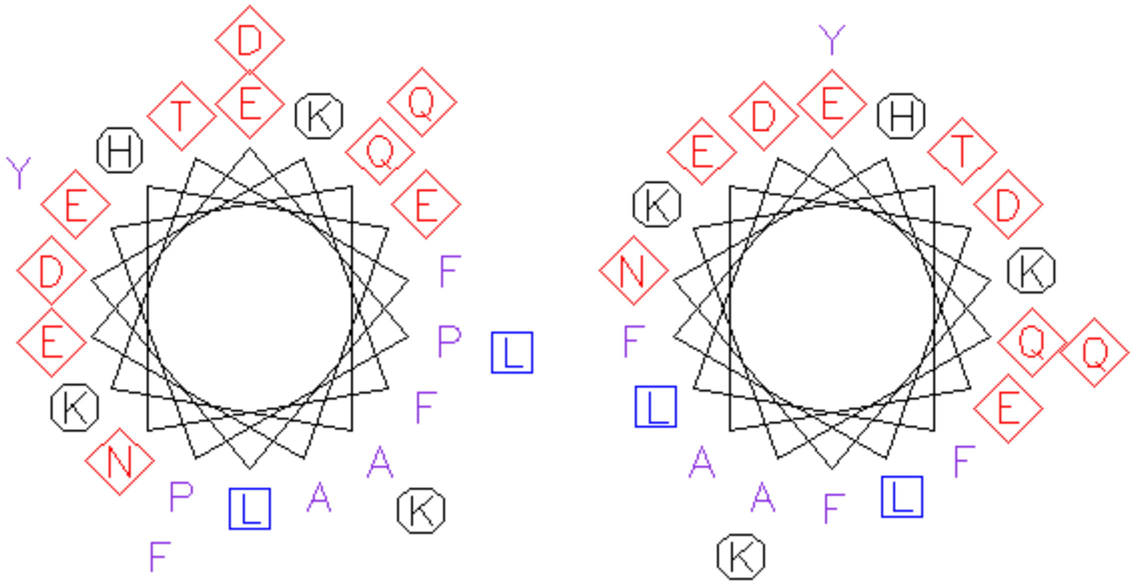

Figure S26: Left: Helical Wheel of P-2-2; Right: Helical Wheel of P-2-3.

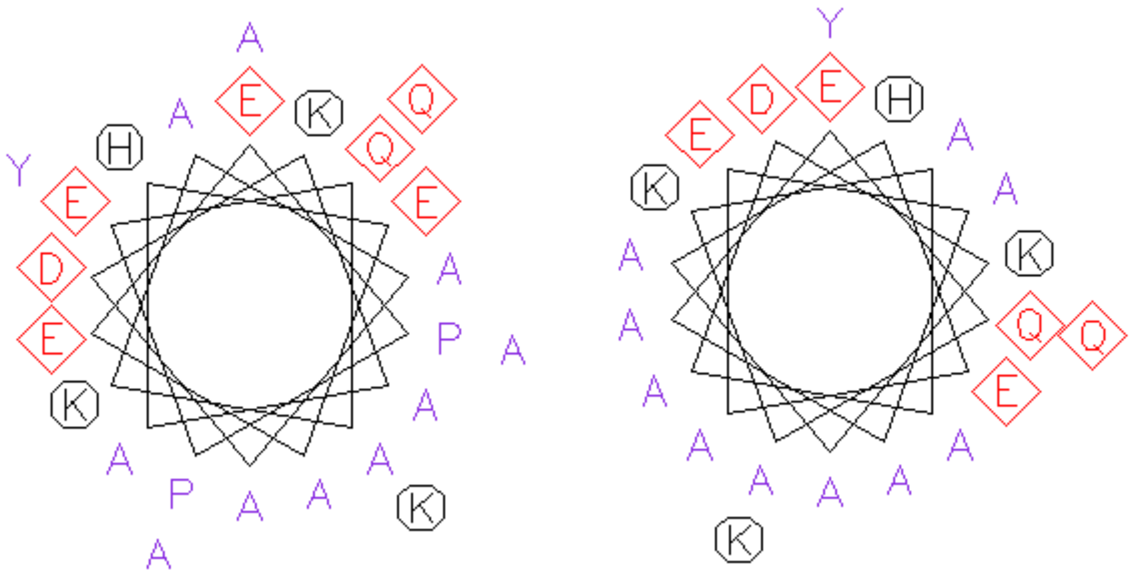

Figure S27: Left: Helical Wheel of P-3-2; Right: Helical Wheel of P-3-3.

5 Binding affinity measurements

5.1 Procedure for binding affinity measurements

For binding affinity measurements, His-tagged wildtype RBD (2019-nCoV) from SARS-CoV-2 (Sinobiological) Spike protein from recombinant expression in mammalian cells was kindly provided by Dr. Coskun (TU Dresden). The provided stock solution was diluted with buffer to 10 µM, rebuffered in sodium carbonate aqueous solution at pH 8.0, and incubated in the dark at 300 µM of the red dye included in the Protein Labelling Kit RED-NHS 2nd Generation from *NanoTemper*. Size exclusion chromatography allowed to obtain the labelled RBD free of unreacted dye. From this concentrated labelled RBD solution a stock of 10 nM was prepared using PBS + 0.05% Tween 20 as diluent.

Microscale thermophoresis (MST) was then measured by making 16 sequential 1:1 dilutions of each peptide, using PBS + 0.05 % (v/v) Tween 20 as diluent, each with a final volume of 10 µL. Each peptide dilution series were then shortly incubated with 10 µL of the 10 nM labeled RBD stock solution, therefore always keeping a 5 nM concentration of the labeled target protein in every sample. MST measurements were conducted at 22 °C with a Monolith NT.115 Pico instrument (*NanoTemper* Technologies), at an excitation power of 20% and a MST power of 40%, the signal was evaluated 1.5 seconds after start of the infrared laser. These conditions were kept constant for all samples. All measurements were performed at least by triplicate. Peptides P-1-1, P-1-2, P-1-3, P-2-3, P-3-3 did not give any significant response in the MST measurements and were considered as “non-binding”. Accordingly no data can be provided in these cases.

5.2 MST binding affinity curves

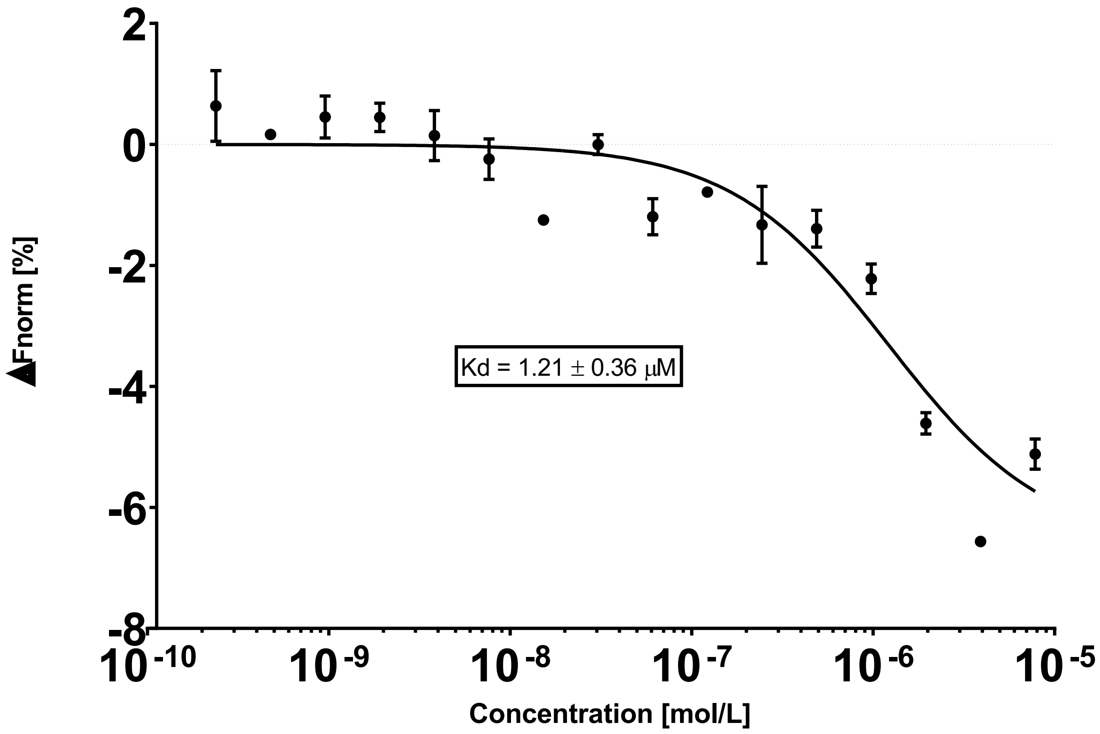

Figure S28: Binding curves for P-2-1 against SARS-Cov2 Spike protein RBD.

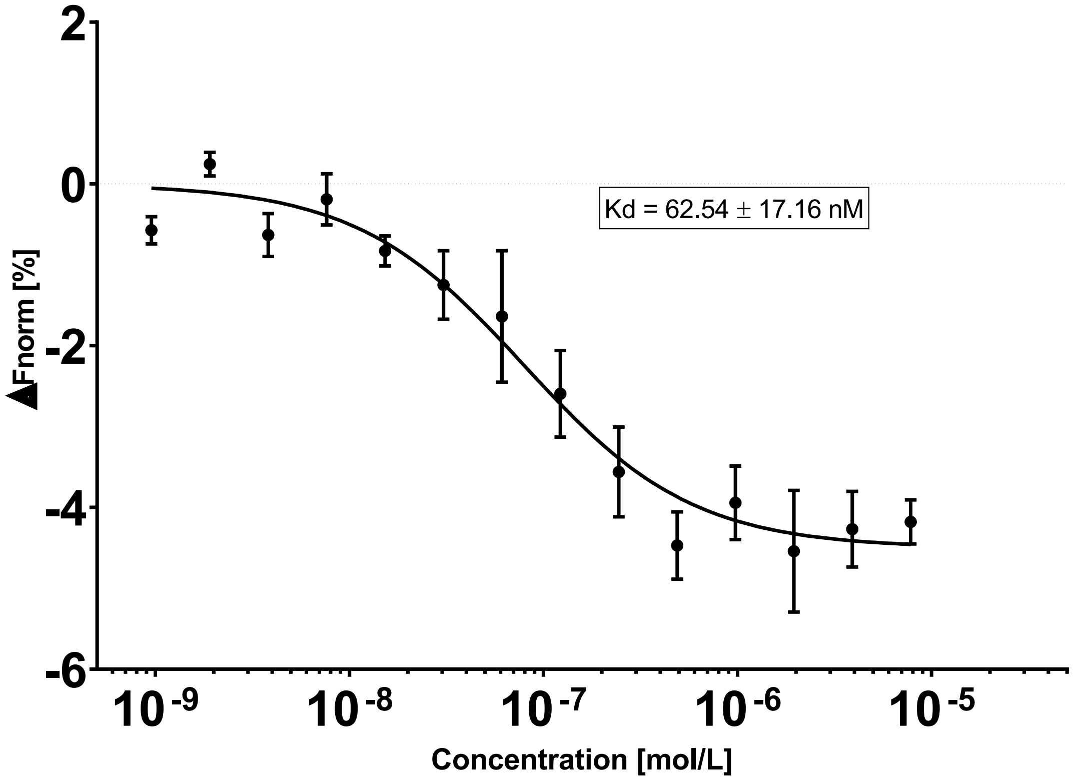

Figure S29: Binding curves for P-2-2 against SARS-Cov2 Spike protein RBD.

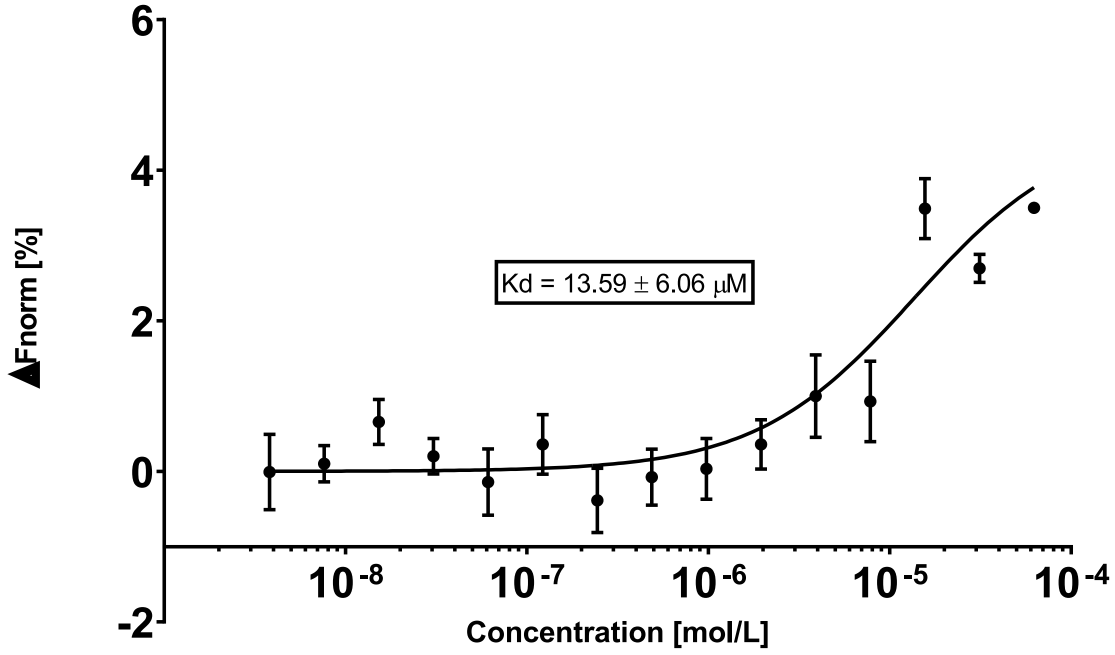

Figure S30: Binding curves for P-3-1 against SARS-Cov2 Spike protein RBD.

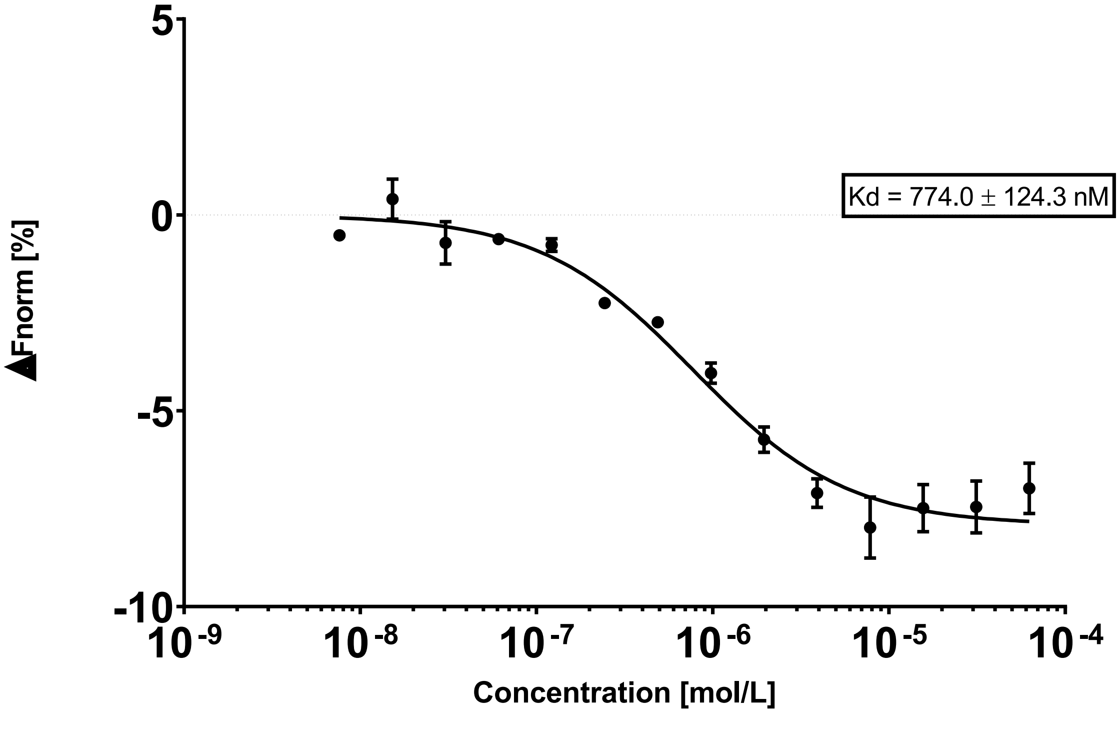

Figure S31: Binding curves for P-3-2 against SARS-Cov2 Spike protein RBD.

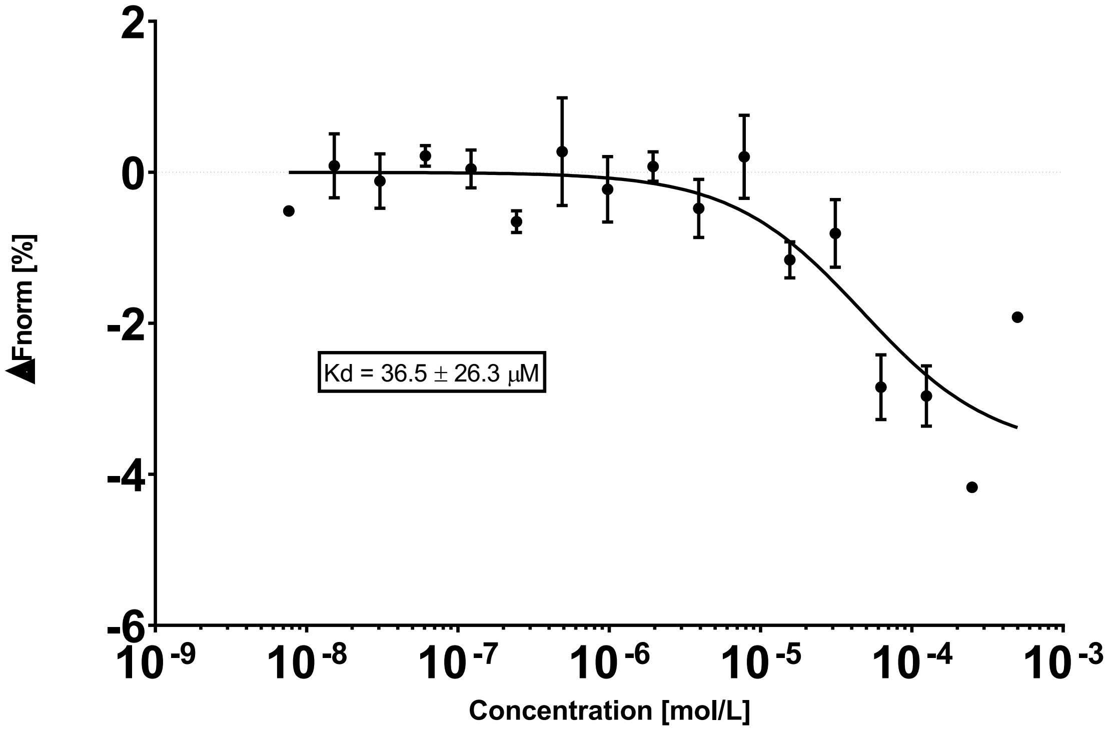

Figure S32: Binding curves for P-4 against SARS-Cov2 Spike protein RBD.

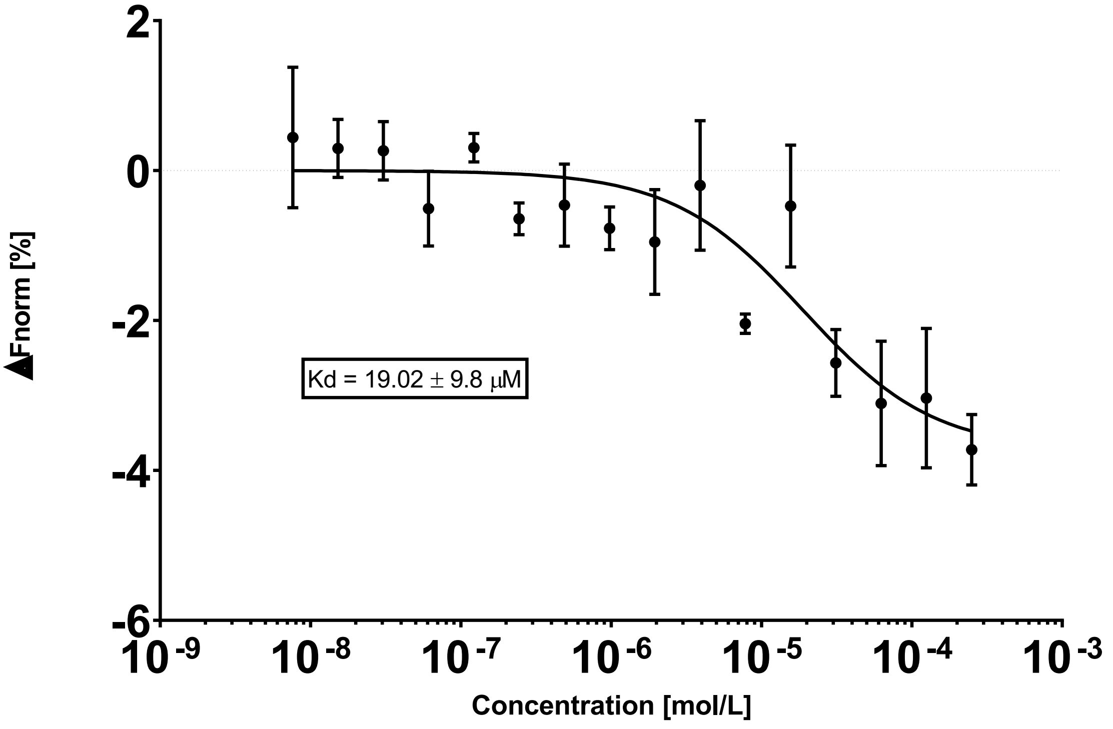

Figure S33: Binding curves for P-5 against SARS-Cov2 Spike protein RBD.

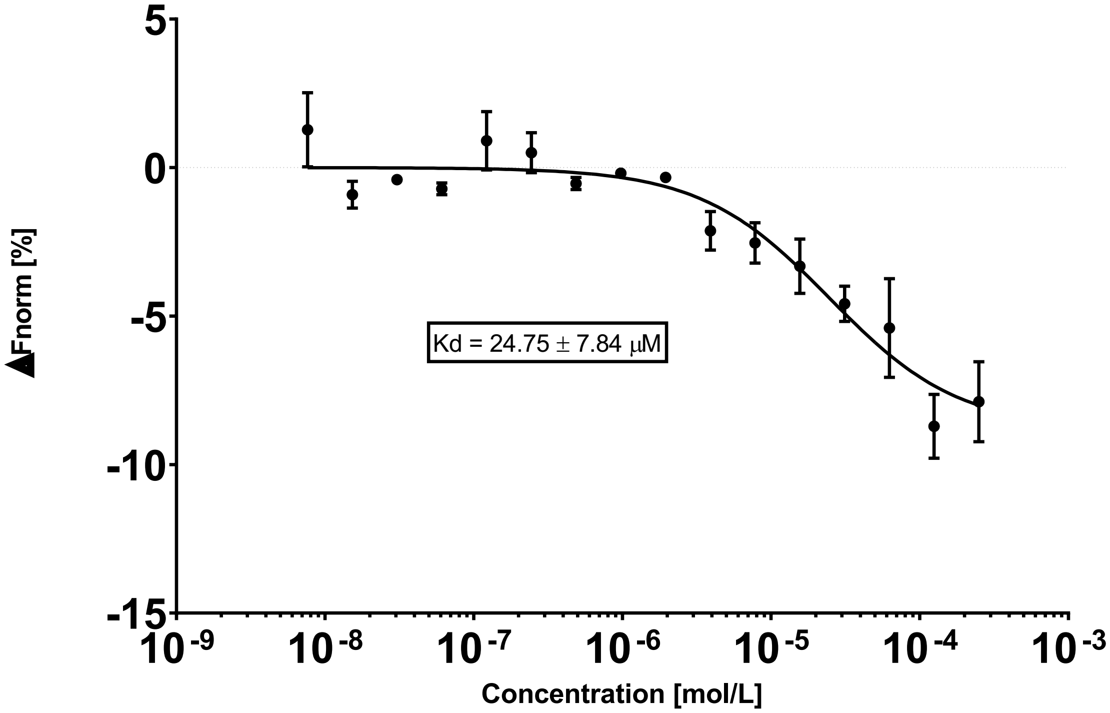

Figure S34: Binding curves for P-6 against SARS-Cov2 Spike protein RBD.

6 References

[1] V. Hack, C. Reuter, R. Opitz, P. Schmieder, M. Beyermann, J.-M. Neudörfl, R. Kühne, H.-G. Schmalz, *Angew. Chemie* **2013**, *125*, 9718–9722.

[2] C. Wiedemann, P. Bellstedt, M. Görlach, *Bioinformatics* **2013**, *29*, 1750–1757.

[3] A. J. Miles, S. G. Ramalli, B. A. Wallace, *Protein Sci.* **2021**, DOI 10.1002/pro.4153.

[4] E. Lacroix, A. R. Viguera, L. Serrano, *J. Mol. Biol.* **1998**, *284*, 173–191.

[5] P. Rice P, I. Longden, B. Bleasby, *Trends Genet.* **2000**, *16*, 276–277.
